# Regulation of regeneration in *Arabidopsis thaliana*

**DOI:** 10.1007/s42994-023-00121-9

**Published:** 2023-11-22

**Authors:** Md Khairul Islam, Sai Teja Mummadi, Sanzhen Liu, Hairong Wei

**Affiliations:** 1 Computational Science and Engineering Program, Michigan Technological University, Houghton, MI 49931 USA; 2 Computer Science, Michigan Technological University, Houghton, MI 49931 USA; 3 Department of Plant Pathology, Kansas State University, Manhattan, KS 66506 USA; 4 College of Forest Resources and Environmental Science, Michigan Technological University, Houghton, MI 49931 USA

**Keywords:** Regeneration, Pluripotency, Transcription factor, Collaborative network

## Abstract

We employed several algorithms with high efficacy to analyze the public transcriptomic data, aiming to identify key transcription factors (TFs) that regulate regeneration in *Arabidopsis thaliana*. Initially, we utilized CollaborativeNet, also known as TF-Cluster, to construct a collaborative network of all TFs, which was subsequently decomposed into many subnetworks using the Triple-Link and Compound Spring Embedder (CoSE) algorithms. Functional analysis of these subnetworks led to the identification of nine subnetworks closely associated with regeneration. We further applied principal component analysis and gene ontology (GO) enrichment analysis to reduce the subnetworks from nine to three, namely subnetworks 1, 12, and 17. Searching for TF-binding sites in the promoters of the co-expressed and co-regulated (CCGs) genes of all TFs in these three subnetworks and Triple-Gene Mutual Interaction analysis of TFs in these three subnetworks with the CCGs involved in regeneration enabled us to rank the TFs in each subnetwork. Finally, six potential candidate TFs—WOX9A, LEC2, PGA37, WIP5, PEI1, and AIL1 from subnetwork 1—were identified, and their roles in somatic embryogenesis (GO:0010262) and regeneration (GO:0031099) were discussed, so were the TFs in Subnetwork 12 and 17 associated with regeneration. The TFs identified were also assessed using the CIS-BP database and Expression Atlas. Our analyses suggest some novel TFs that may have regulatory roles in regeneration and embryogenesis and provide valuable data and insights into the regulatory mechanisms related to regeneration. The tools and the procedures used here are instrumental for analyzing high-throughput transcriptomic data and advancing our understanding of the regulation of various biological processes of interest.

**Supplementary Information:**

The online version contains supplementary material available at 10.1007/s42994-023-00121-9.

## Introduction

Plants have a remarkable capacity for regeneration, which is defined as the physiological renewal, repair, or replacement of tissues or organs (Ikeuchi et al. 2016). Regeneration occurs in plants when they are subjected to various wounds caused by mechanical forces, insect and animal feeding, and some human activities. Despite significant research into this phenomenon in some model organisms, questions remain about the molecular mechanisms at play and regenerative capability. Regenerative capacity is generally conferred by totipotency, pluripotency, and multipotency. Totipotency refers to the regenerative potential of the fertilized cell and the first few rounds of cell divisions after fertilization that can develop into all the cell types in a body, including the extraembryonic cells. Pluripotency refers to the large mass of embryonic cells formed after the first few rounds of cell divisions; pluripotent cells can give rise to all cell types that make up the body. Finally, multipotency refers to cells that can develop into more than one cell type but are more limited compared to either totipotent or pluripotent cells, for example, stem cells in apical or lateral meristems.

During plant development, shoot apical meristems (SAM), root apical meristems (RAM), and lateral meristems contain stem cell masses that are responsible for continuous growth and development by differentiating into various specialized cell types. When plants are wounded or specific tissues or organs are cut and cultured on callus induction medium (CIM), certain combinations of autogenous or externally applied plant hormones, such as auxins and cytokinins, can induce de-differentiation at wound sites. These hormones help initiate cellular reprogramming and enable specific differentiated cells to revert to an undifferentiated state, including those in callus and meristematic tissues. In an undifferentiated state, cells gain regenerative capability and can differentiate into new tissues, organs, or even whole plants (Ikeuchi et al. 2016). The exact molecular mechanisms underlying plant regeneration remain largely elusive and may vary with the tissue type that is wounded. For a while, it was envisaged that some pluripotent/multipotent cells or dormant buds ‘lurk’ in plant tissues, and that when plants are wounded, these cells or buds are activated leading to the development of new tissues, organs, or entire plants. However, there is little evidence that supports this claim.

Some recent studies in animal limb regeneration (Gerber 2018) and plant adventitious rooting (Wang et al. 2022) suggest that some types of cells at wound sites revert to undifferentiated states and regain pluripotency or multipotency upon wounding, allowing them to develop into a new tissue, and organ. Somatic embryogenesis is also an important biological process for plant regeneration. In somatic embryogenesis, a somatic cell undergoes de-differentiation, forms an embryo-like structure, and ultimately develops into a whole plant. In contrast, an embryo that has developed the stem cell-like characteristics and the ability to regenerate can produce a whole plant when the seed germinates. This process involves a complex interplay of transcription factors (TFs), hormonal signaling pathways, and epigenetic regulation (Zimmerman 1993).

Under natural conditions, plant regeneration is influenced by endogenous plant hormones, such as auxins and cytokinins, and the inherent ability of specific cells to de-differentiate and subsequently differentiate into new tissues, organs, or whole plants. Researchers have imitated this process in the laboratory using CIM to induce callus regeneration; the calli are then transferred to shoot induction medium (SIM) and root induction medium (RIM) to facilitate the regeneration of the shoots and roots, respectively. In general, roots, shoots, leaves, whole explants, and hypocotyls are initially cultured in an auxin-rich CIM to stimulate callus formation. Subsequently, these calli are transferred to a medium rich in cytokinins, known as SIM, to induce shoot regeneration. In addition, callus cells or shoot explants can be transferred to a medium with a higher concentration of auxins relative to cytokinins, called RIM, for root generation (Kang et al. 2022). Therefore, if we could gather high-throughput RNA-seq data generated under the same or similar regeneration conditions to those described above, we could use them to identify the major regulatory genes that control regeneration.

Computational methods have been widely used to analyze omics data, including the transcriptome, proteome, and metabolome, to identify key players and underlying molecular mechanisms to advance our understanding of plant growth and development. For example, using bioinformatics approaches, such as gene identification using the Hidden Markov Model, phylogenetic relationships, motif prediction, and tissue-specific expression profile analysis, Hao et al. discovered that *GmWOX18* in soybean enhanced the regeneration capability of clustered buds (Hao et al. 2019). In addition, using Illumina Digital Gene Expression (DGE)-based gene expression profiling, and biochemical and histological approaches, Yang et al. (2012) revealed the molecular mechanisms involved in somatic embryogenesis in cotton were identified. In another approach in cotton, Sun et al., focused on PCA rather than DEGs and identified 46 transcripts that may contribute to the transformation from a non-embryogenic to an embryogenic callus (Sun et al. 2018). PCA showed homogeneity in embryogenic callus samples, suggesting that these samples have similar gene expression patterns and are likely to have similar regeneration potential. In *Arabidopsis thaliana* (*A. thaliana*), Horstman et al. performed ChIP-seq analysis to identify the binding sites of BBM transcription factor in somatic embryo tissue (Horstman et al. 2015). In a follow-up study (Horstman et al. 2017), they found that BBM transcriptionally regulates LEC1, LEC2, FUSCA3 (FUS3), and ABSCISIC ACID INSENSITIVE3 (ABI3). LEC2 and ABI3 quantitatively regulate BBM-mediated somatic embryogenesis. Finally, using transcriptomic data and our top-down GGM algorithm, we identified PuHox52, and its two target genes, PuMYB40 and PuWRKY75, which collectively regulates adventitious rooting at the basal ends of *Populus ussuriensis* stem cuttings (Wang et al. 2022; Wei et al. 2020).

In our study, we explored public repositories and selected transcriptomic data that were primarily generated from *A. thaliana* leaves, roots, shoots, whole explants, and hypocotyls on CIM, which have an inherent capacity to de-differentiate into calli when cultured on CIM. We also collected data that were generated from callus, SAM, and RAM, which have stem-cell-like properties and the capacity to differentiate into various specialized cell types and tissues. Then, we gathered 145 previously identified key TFs that have a role in regeneration or related processes. These previously identified TFs were used to guide computational analysis to identify other key player genes involved in regeneration, according to the procedure that follows. First, we built collaborative subnetworks of all TFs using CollaborativeNet in two steps: (1) construction of a Shared Co-expression Connectivity Matrix (SCCM) (Nie et al. 2011), which codes a collaborative network of all TFs; (2) decomposition of the SCM into multiple subnetworks using a heuristic-based triple-link algorithm and a Compound Spring Embedder (CoSE)-based layout algorithm; each subnetwork contained a certain number of TFs that were highly collaborative with each other, but less frequently collaborative with TFs in other subnetworks, indicating that they collaborated to regulate a biological process or pathway. This analysis resulted in nine collaborative subnetworks of interest. To study these subnetworks, PCA was carried out, and the first principal component (PC1) and the gene expression levels were plotted together to judge how much the gene expression values could explain the principal components. Among the nine subnetworks, subnetworks 1, 12, and 17 were selected for further Gene Ontology (GO) analysis; somatic embryogenesis (SE) (GO:0010262) and regeneration (GO:0031099) were statistically over-represented in the CCGs of Subnetwork 1 but not in the CCGs of Subnetworks 12 and 17. Analysis of binding motifs of known TFs in these three subnetworks in the proximal promoters of their CCGs was carried out. Finally, the Triple-Gene Mutual Interaction (TGMI) algorithm was applied to TFs in Subnetwork 1 and its CCGs involved in somatic embryogenesis (GO:0010262) and regeneration (GO:0031099) to rank the TFs based on their interference frequencies with genes involved in these two biological processes. We also conducted TGMI for TFs in Subnetwork 12 based on their interference frequencies with genes involved in embryo development (GO:0009790) and for TFs in subnetwork 17 based on their interference frequencies with genes involved in embryo development (GO:0009790) and callus formation (GO:1990110). Our comprehensive analyses identified some novel TFs that may have regulatory roles in regeneration and embryogenesis. However, it is important to validate these in silico findings experimentally in the future. These results provide valuable insights into the gene regulatory networks and mechanisms related to regeneration. Furthermore, the tools and the procedures utilized in this study will be instrumental for analyzing high-throughput transcriptomic data and advancing our understanding of the regulation of various biological processes of interest.

## Methods and materials

### Data collection

Considering the aim of the study, we collected transcriptomic data (RNA-seq) from the Sequence Read Archive (SRA) database, National Center for Biotechnology Information (NCBI). In our research, we utilized NCBI’s user interface to query transcriptome data (RNA-seq) generated from de-differentiation and re-differentiation-related processes, for example, callus induction from various tissues and differentiation of callus and explant tissues (stems or leaves) into SAM and RAM (Leinonen et al. 2011). The goal of our research is to identify regulators that control regeneration capability in various organs of *A. thaliana* plants. Therefore, we searched for keywords, such as callus, totipotency, pluripotency, SAM, and RAM, to identify RNA-seq data that contains information enriched for regeneration, pluripotency, and multipotency maintenance. Moreover, we considered other tissues like leaves, shoots, roots, whole explants, and hypocotyls, some of which were induced in CIM enriched with hormones (e.g., auxins). We queried the SRA database by combing these words with and tissues. Finally, manual curation was performed to obtain the data that met our requirements.

Metadata was obtained via NCBI’s specialized user interface functions. We evaluated sample properties including cell and tissue types, and experimental methods for ultimate inclusion in the study. We used the Unix operating system to download and preprocess the data. Fastq-dump, a utility bioinformatics tool in NCBI’s SRA toolkit, was employed to download transcriptome data with the SRA accession identifiers of RNA-seq samples (https://edwards.flinders.edu.au/fastq-dump/). A Python wrap was developed and used to call fastq-dump to download all data sets recursively.

### Data preprocessing

The quality of each RNA-seq data file was examined using FASTQC, a tool specifically designed for checking the quality of high-throughput sequence data (https://www.bioinformatics.babraham.ac.uk/projects/fastqc/). Given that some datasets exhibited quality issues, we employed Trimmomatic to remove adaptor sequences and low-quality reads from high-throughput sequencing data (Bolger et al. 2014). Python programming was used to empower both of these tools by feeding the input files and handling the output files.

STAR, a sequence aligner (Dobin et al. 2013), was employed for mapping the reads to the genome reference of *A. thaliana*. Genome sequence and annotation files, including General Transfer Format (GTF), of *A. thaliana* TAIR10 were downloaded from Phytozome (Goodstein et al. 2012). First of all, genomic index files were created using genome sequence, GTF file, and Bowtie (https://bowtie-bio.sourceforge.net/index.shtml). Then, the alignment of the sample’s reads was performed by supplying the index files and obtaining output files in BAM format using STAR. Finally, Cufflinks was used to assemble the individual transcripts from the reads aligned to the genome and to calculate expression levels for all splicing variants of each gene (Trapnell et al. 2010). In our case, the expression levels were measured by a normalized form of values named FPKM returned by the program. Before performing further analyses, we dropped the genes that had more than 92% zero values across samples.

### Construction of collaborative subnetworks of regeneration TFs

In our study, we employed a method named CollaborativeNet (https://github.com/hwei0805/TF_ CollaborativeNet) (Ji et al. 2017; Nie et al. 2011), initially known as TF-Cluster, to build a collaborative network of all TFs, which was subsequently decomposed into collaborative subnetworks using the Triple-Link algorithm integrated within CollaborativeNet package. Each subnetwork was proven to regulate a biological process as evidenced by the examples shown in original publications and some other subsequent studies. For further verification of the subnetworks, we also applied Compound Spring Embedder (CoSE), a layout algorithm that was developed by other groups, to generate the subnetworks (Dogrusoz et al. 2009). CoSE is a force-directed graph-based layout algorithm that effectively positions the nodes and edges in a network, providing a visually appealing representation of the underlying structure. It was necessary that the pairwise relationships of all TFs in the SCCM have at least 25 shared CCGs. We first extracted the number of shared CCGs from the SCCM matrix, and then used the cytoscape’s CoSE layout to visualize the subnetworks while increasing the distance among likely subgroups and, finally, removing the connections among clearly distinguishable subgroups.

### Identifying subnetworks that potentially regulate regeneration

We performed PCA analysis to identify the most pertinent collaborative subnetworks to regeneration, each characterized by strong collaborative concordances. We plotted the PC1 and the gene expression of TFs for each subnetwork, where the PC1 explained the variances across multiple tissue types. By analyzing the peaks and variation of PC1, and expression profiles in 78 RNA-seq samples from multiple tissues, we were able to assess whether the expression values played a significant role in shaping the PC1 in each specific tissue type. This analysis greatly aided in our assessment of whether a specific subnetwork may exert significant influence over pluripotency and multipotency. In addition to PCA analysis, we also evaluated the gene functions of transcription factors (TFs) within each subnetwork by leveraging existing annotations.

### GO analysis and construction of GO trees

We used ShinyGO V0.76 (Ge et al. 2020) to identify the enriched GO terms in the CCGs associated with each selected collaborative subnetwork. The cut-off threshold for statistically significant GO terms was set to 5 × 10^*−*2^ for the corrected *P* values. The enriched GO terms representing the various biological processes related to pluripotency or multipotency, for example, regeneration, somatic embryogenesis, callus formation, and embryo development, were studied. The enriched GO terms were used to construct GO trees with ‘Ancestor Chart’ tool available at from EMBL-EBI (https://www.ebi.ac.uk/) to view the hierarchical locations of nodes of interest, especially, the parent and child nodes, so that their relevance to pluripotency or multipotency can be determined.

### Motif search

Motif enrichment analysis was used to identify over-represented transcription factor binding sites (TFBSs) in a set of CCGs. We used the data within CIS-BP Database, where TFBSs were obtained from experimental validation using techniques such as Protein-Binding Microarray experiment or chromatin immunoprecipitation (ChIP). Once a binding site of a TF was identified, a PWM was constructed based on the alignment of known instances of the binding site. This database includes the DNA sequence motifs and the corresponding transcription factors that recognize them (Weirauch et al. 2014). Motif scanning was performed on the proximal promoter regions of 2000 basepairs (bps) of the CCGs for a TF using MotifLocator (Claeys et al. 2012), and the output includes a list of potential binding sites, along with their scores and locations in the input sequence. In the end, a comparison of the occurrence of the motifs in the CCGs in parallel with that of the whole genome set was conducted using hypergeometric test. We conducted the test to determine the probability of observing the specified number of TFBSs within the CCGs of each TF from the subnetwork by chance given the total number of TFBSs in the genome. Lower p-values indicate an enrichment of the TFBS of a TF and the CCGs, and vice versa.

### Identification of TFs that interfered with the regeneration-related biological processes

We also utilized the Triple-Gene Mutual Interaction (TGMI) algorithm (Gunasekara et al. 2018) to validate and rank potential regulators identified by CollaborativeNet based on their regulatory interference with the biological processes, such as regeneration, somatic embryogenesis, callus formation, and embryo development. This algorithm assesses the regulation potentials among combined blocks of three genes: one TF from a subnetwork and two CCGs of this subnetwork that are known to be involved in one of the above-mentioned biological processes. The extent of regulation is determined by the mutual interaction measure (MIM), which quantifies the regulatory strength exerted by the TF on the two biological process genes (CCGs) (Gunasekara et al. 2018). Triple gene blocks with a false discovery rate (FDR) below the significance threshold of 0.05 were considered to possess a significant MIM, and each TF’s interference with each CCG was counted once. After evaluating all possible triple-gene combinations, we ranked the TFs within each subnetwork based on how frequently each of them interfered with the CCGs associated with a specific biological process, referred to as interference frequency, which reflects the overall regulatory potential of a TF in controlling a specific biological processes. We also compared TF interference frequency with the motif search results to evaluate the possibility of each TF regulating regeneration and embryogenesis-related processes.

## Results

### Data acquisition and processing

In our study, we initiated the data collection process by gathering the run-identifiers (Run-ID) and metadata associated with RNA-seq samples from A. thaliana. From this initial dataset, we carefully selected samples that captured crucial moments in de-differentiation or re-differentiation processes. We identified 78 samples to be included in our data after manually curating and filtering them based on several criteria (Supplemental Table 2). Among them, twenty-two and six samples were taken from stem cell mass regions called SAM and RAM, respectively. Seven samples were taken directly from calli. In addition, we included four leaves, four root, thirteen whole explant, eleven shoot, and eleven hypocotyl samples, all of which were cultured in CIM. These samples covered the entire processes, starting from de-differentiation into calli from a specific tissue and re-differentiation into shoots or roots from calli in cytokinin-rich or auxin-rich media, respectively.

RNA-seq data files in FASTQ format were downloaded from the SRA database, NCBI, and were subjected to quality control and trimming procedures on the sequencing reads. Next, the cleaned reads were aligned to the *Arabidopsis* TAIR 10 reference genome, and transcripts were assembled and quantified. The result is a set of FPKM values (Fragments Per Kilobase of Transcript per Million mapped reads) for each transcript, which provides normalized expression levels considering both sequencing depth and gene length.

Then, we conducted an extensive literature review to collect information on known transcription factors (TFs) that regulate pluripotency, multipotency, and regeneration with an assumption that pluripotency and multipotency are essential for regeneration. We identified a total of 145 genes, including 78 TFs and 67 non-TFs, from literature (Supplemental Table 1). These genes are known to play a role in pluripotency and multipotency maintenance and regeneration process. To visualize the expression patterns of these 145 pluripotent genes across the 78 samples, we generated a heatmap (Supplemental Fig. 1). The details on the origin of these 78 samples collected from *A. thaliana* are shown in Supplemental Table 2. This information is important for understanding the tissue specificity and functions of the identified TFs and non-TFs, as well as their roles in various developmental stages during the regeneration process.

### Collaborative- and CoSE-based collaborative subnetwork construction

The goal of our study is to identify the TF genes that play pivotal role in pluripotency, multipotency, or the regeneration of whole plants from a single cell. In this context, pluripotency and multipotency refer to the ability of a cell to differentiate into nearly all or some cell types found within an organism, respectively. This capability is instrumental in facilitating the regeneration of various tissues and organs (Verdeil et al. 2007). Pluripotency and multipotency in different cells have been shown to be regulated by a number of genes and molecular pathways. These include genes associated with the renewal of pluripotency, cell division, differentiation, and epigenetic regulation (Fehér 2019). One of the key factors that regulates pluripotency and multipotency in *A. thaliana* is the presence of specific TFs that control the expression of genes involved in cell division and differentiation. An example is the WUSCHEL (WUS) gene, a pivotal regulator of stem cell maintenance in *A. thaliana*, which is a key regulator of stem cell maintenance in *A. thaliana* and is required for the regeneration of whole plants from somatic embryos (Schoof et al. 2000).

**Fig. 1:**
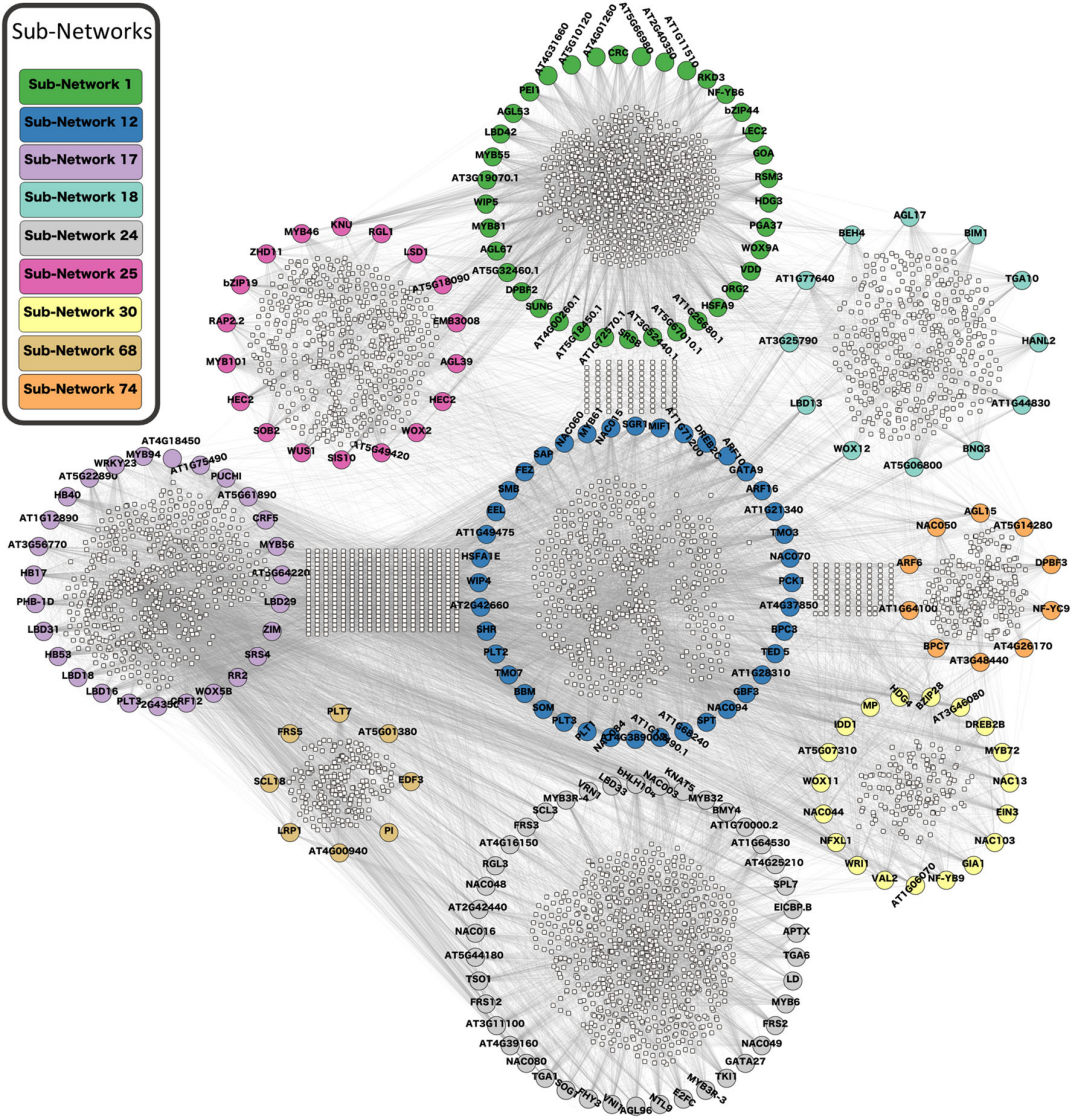
Nine collaborative subnetworks and their co-expressed and co-regulated genes (CCGs) identified using the CollaborativeNET method (referred to previously as TF-Cluster). Each of the nine subnetworks is distinct and consists of collaborative transcription factors (labeled nodes) and CCGs (small nodes) discovered by the CollaborativeNET algorithm. The CCGs within each circular subnetwork pertain to that subnetwork, while the CCGs not within any circular subnetwork are shared by two or more subnetworks

**Fig. 2:**
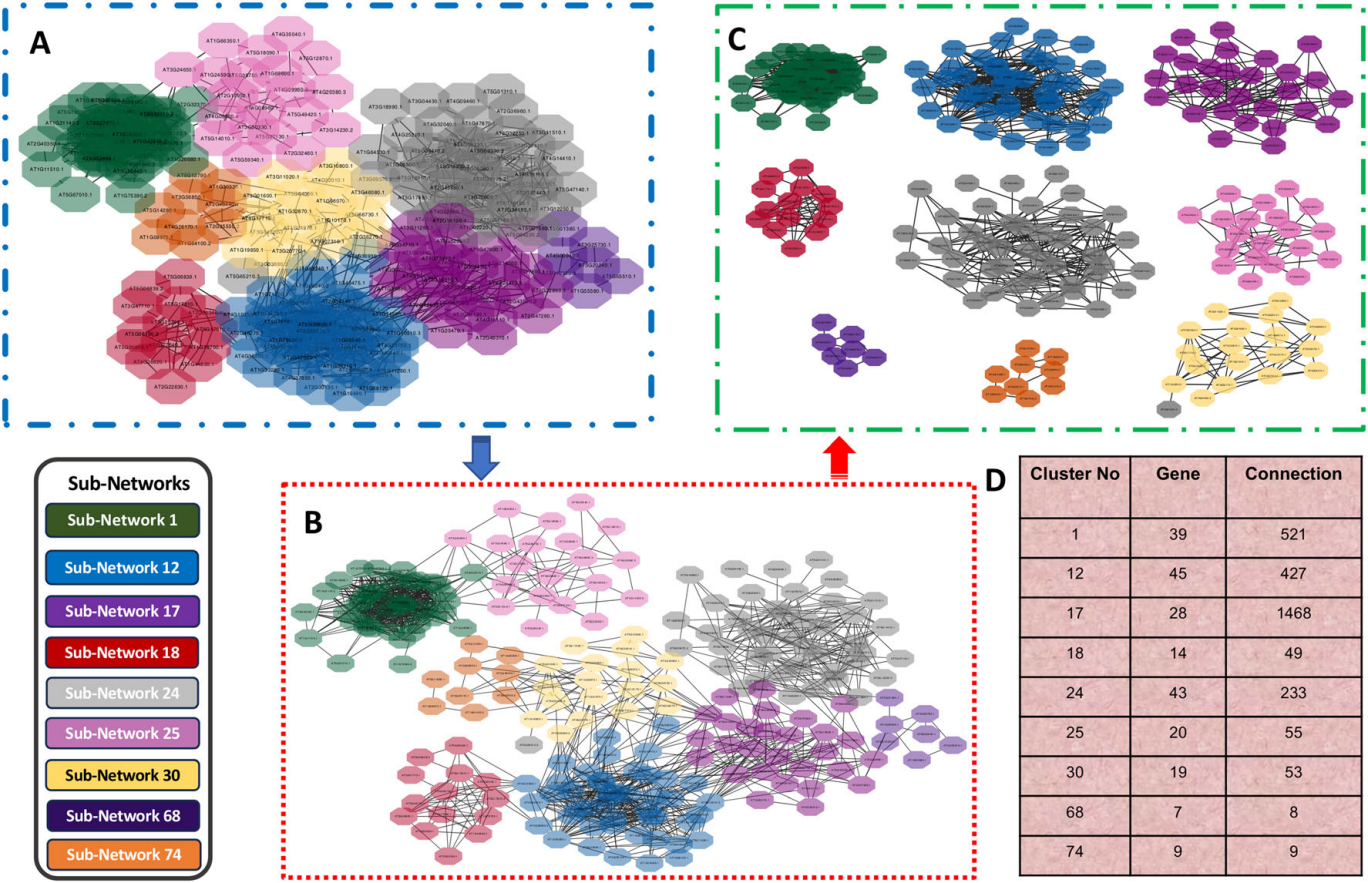
The development of collaborative subnetworks using the Compound Spring Embedder (CoSE) Layout algorithm. Each subnetwork is distinguished by a distinct color as shown. **A** The initial depiction of the network revealed multiple subnetworks, highlighting groupings of interacting transcription factors (TFs). **B** A more refined and segmented visualization of subnetworks. **C** An isolated representation of the subnetworks. **D** The subnetworks and the connections among the TFs in each subnetwork

In this study, we utilized CollaborativeNet to construct a comprehensive collaborative network encompassing all transcription factors (TFs). Initially, all 1622 known TFs were used to build SCCM, which held all collaborative regulatory gene pairs. The SCCM was then decomposed into 280 non-overlap subnetworks (Supplemental Table 3) using a Triple-Link algorithm in the CollaborativeNet package. It's important to observe that subnetworks created earlier exhibited a higher number of high collaborative edges compared to those generated later in the decomposition process, and that the subnetworks generated after the initial 100 were generally smaller and featured weaker collaborative strengths in comparison to their predecessors (Supplemental Fig. 2). This is because the Triple-Link algorithm always chooses two TFs with the highest concordance of collaboration in the SCCM to decompose it. We studied the functional annotation of TFs in each subnetwork with respect to the regeneration. Thus, we mapped the 78 known pluripotent TFs (Supplemental Table 1) to facilitate the identification of crucial subnetworks. We also incorporated existing gene annotations in *A. thaliana* to identify crucial subnetworks. Our manual curation, guided by domain knowledge, allowed us to narrow down the number of subnetworks to nine subnetworks. Table 1 shows the number of TFs in each subnetwork along with the number of regeneration-related key pluripotency genes identified from literature, as shown in Supplemental Table 1. The nine subnetworks and their collaborative relationships are displayed in Fig. 1. It is evident that subnetworks 1, 12, 17, and 74 had a high number of shared CCGs. In contrast, other subnetworks shared fewer common CCGs, which were not shown in the Fig. 1. Furthermore, subnetworks 1, 12, and 17 had more intra-connections, indicating that the number of common CCGs between any given paired TFs within each subnetwork was generally higher for these three subnetworks compared to others. We also applied the force-directed layout algorithm called CoSE to the SCCM and found that the nine subnetworks are separately recognizable with different colors (shown in Fig. 2A). Moreover, subnetworks 1, 12, and 17 had the highest intra-connections among TFs compared to other subnetworks. It is noteworthy to mention that the subnetworks decomposed by the CoSE algorithm were almost the same as Triple-Link algorithm. Only one TF, AT5G65210.3, which was in Subnetwork 24 in the output of Triple-Link algorithm, was decomposed into Subnetwork 30 in the output of CoSE.

**Table 1:** Summary of the selected subnetworks that were found from shared co-expression connectivity matrix (SCCM) cluster analysis

Subnetwork no	Number of transcription factors	Key pluripotency genes	Gene names
1	39	3	*LEC2*, *WOX9*, *PGA37*
12	45	8	*PLT1*, *PLT2*, *ARF10*, *SCR*, *ARF16*, *SHR*, *BBM*, *PLT4*
17	28	6	*LBD18*, *LBD16*, *WOX5*, *LBD29*, *PLT3*, *BRAVO*
18	14	1	*WOX12*
24	44	2	*LBD17*, *BAM7*
25	20	3	*ESR2*, *WUS*, *ABI3*
30	19	4	*LEC1*, *VAL2*, *HSL1*, *ERF115*
68	8	2	*LAS*, *PLT7*
74	10	1	*AGL15*

The third column shows the number of crucial genes for pluripotency that has been demonstrated in earlier studies

**Fig. 3:**
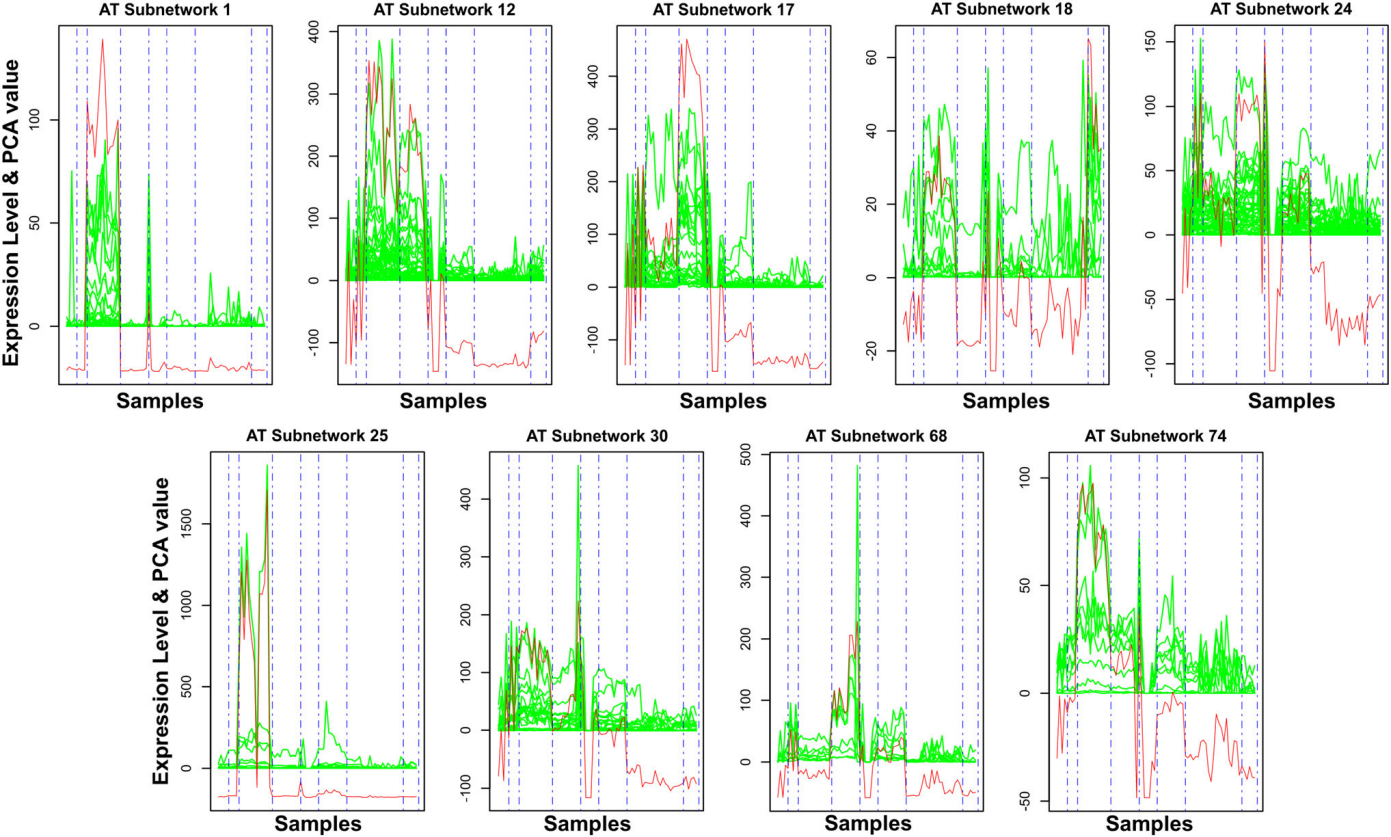
Gene expression levels and principal component analysis (PCA) of nine collaborative subnetworks of *Arabidopsis thaliana*. The expression level of each gene is represented by the green curve, and the first principal component (PC1) of each subnetwork is represented by the red curve. Different tissues are arranged in a specific order from left to right, as follows: leaves, roots, whole explants, hypocotyls, calli, shoots, SAM, and RAM. Tissues to the left of callus were under de-differentiation toward the callus while tissues to the right of callus were under differentiation from callus to shoots and roots, including SAM and RAM

In Fig. 3, a comparison of the expression values of TFs within each of the nine subnetworks and PC1 was presented across different tissue groups. The expression patterns of genes within each subnetwork were depicted with green lines, while the expression of PC1 was represented by red lines across all the tissues. Vertical blue dashed lines were used to demarcate specific tissue types. The primary purpose of Fig. 3 is to visually compare the gene expression values of the given subnetworks with the PC1. A high consistence peak between expression values and PC1 indicates the principal components can be predominantly explained by the expression values irrespective of the signs. The sign of PC1 could be either positive or negative, depending on the covariance between the input features, which corresponds to the directions of the vectors in the new coordinate system defined by the first principal component. The sign is arbitrary and can be flipped without changing the underlying relationships in the data. The analysis revealed that the gene expression trends were similar to PC1 in Subnetworks 1, 12, 17 and 74 across multiple tissue samples, particularly in the samples from the de-differentiation phase (callus induction process from differentiated tissues) where the PC1 was mostly positive, indicating the PC1 represents the directions of the data that explain a maximal amount of variance. Conversely, negatively directed PC1 was observed in multiple samples including callus, shoots, SAM and RAM (differentiation of callus into different tissue) in most subnetworks. Subnetworks 1, 12, 17, and 74 had high expression peaks and PC1 values in the de-differentiation process, and therefore, were more likely to exert strong regulation on their targets (e.g., some CCGs) during regeneration. In contrast, other subnetworks displayed more an arbitrary pattern (no single dominant trend) in both PC1 and gene expression, indicating more complex biological functions or heterogeneous patterns among the genes. The similarity in trends between gene expression and PC1 within each subnetwork within each subnetwork implies that these genes may be co-regulated or function together in related biological processes. Based on these findings and gene annotation, subnetworks 1, 12, 17, and 74 were selected for further GO analysis.

### GO analysis and results

**Fig. 4:**
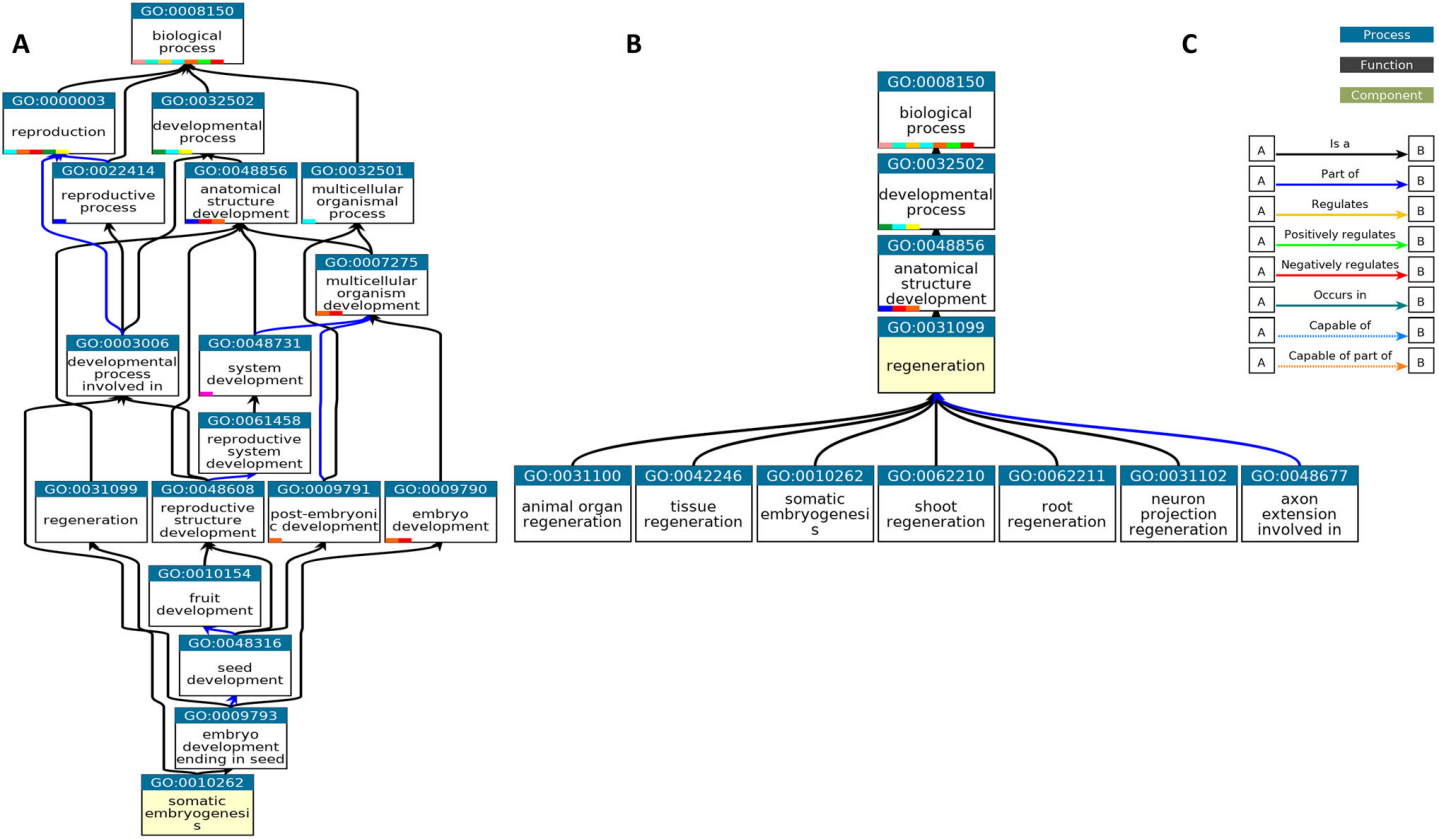
Visualization of the hierarchical relationships among gene ontology (GO) terms through a directed acyclic graph (DAG). The DAG captures the complex structure of ancestors and descendants of GO terms related to somatic embryogenesis and regeneration processes over-represented by the co-expressed and co-regulated genes (CCGs) of Subnetwork 1, providing valuable insights into the functional organization and dependencies within the GOs. **A** Directed Acyclic Graph (DAG) for somatic embryogenesis (GO:0010262), **B** regeneration (GO:0031099). **C** Legend

**Fig. 5:**
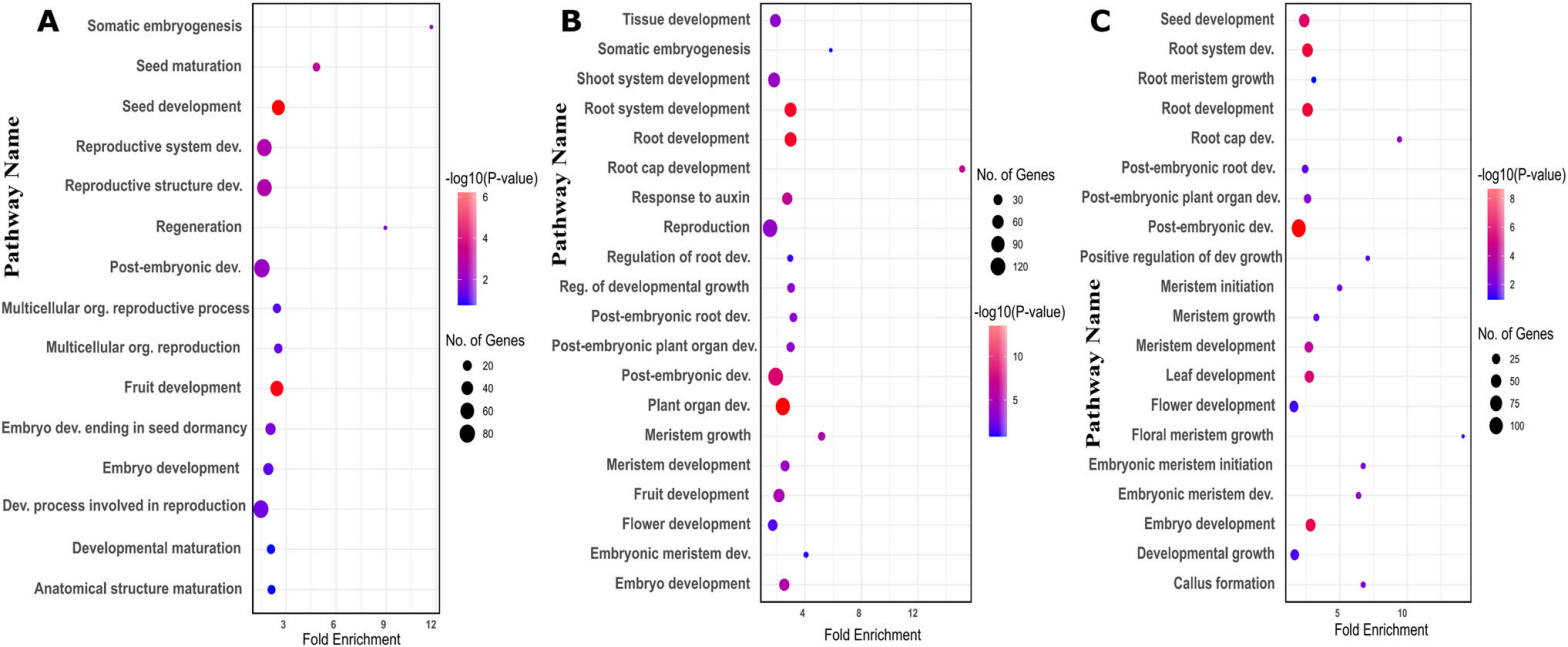
Gene ontology (GO) enrichment analysis performed with the co-expressed and co-regulated genes (CCGs) associated with each subnetwork using ShinyGO V0.76. Each enriched GO term is represented by a dot plot. **A** GO enrichment results of CCGs associated with Subnetwork 1, **B** GO enrichment results of CCGs associated with subnetwork 12, and **C** GO enrichment results of CCGs associated with subnetwork 17

Since most TFs in a subnetwork are not well characterized and annotated in functions, we merged the top 100 CCGs of each TF for each given subnetwork and then removed redundant ones for inferring the enriched functions terms that can present the primary function of the subnetwork. This can be accomplished by GO enrichment analysis. For example, after merging the CCGs of 39 TFs in subnetwork 1 and removing the redundant ones, 980 unique CCGs were obtained and used for GO enrichment analysis. The importance of GO analysis lies in its ability to provide the enriched biological processes. By analyzing the enrichment of GO terms representing biological processes and their hierarchical relationships, researchers can gain a better understanding of the biological processes that are active in a particular tissue. We conducted GO analysis with the unique CCGs associated with each of the three subnetworks using ShinyGO V0.76 (Ge et al. 2020) and established a threshold of 0.05 for corrected *P*-values to identify the most significant GO Terms. 20, 131, 313, and 141 enriched GO terms were obtained for subnetworks 1, 12, 17, and 74, respectively. The most significant GOs associated with regeneration are displayed in Fig. 4. Somatic embryogenesis and regeneration, two GOs critical for regeneration, were enriched in Subnetwork 1 with adjusted *P* values of 0.007 and 0.02, respectively (Supplemental Table 4). Subnetworks 12 was dominated by the GO terms that represent root system development (Supplemental Table 5), while subnetwork 17 was enriched with the GO terms of root/shoot/floral meristem growth and callus formation (Supplemental Table 6). We also implemented GO analysis to subnetwork 74’s CCGs but failed to identify GO terms relevant to regeneration (Supplemental Table 7). But there were some tissue-specific regeneration processes like leaf formation, seedling development, regionalization, and meristem structural organization. Subnetworks 1, 12, and 17 are obviously more relevant to the regeneration process, as evidenced by the prominent GO terms shown in Fig. 5.

### CCGs of in subnetwork 1, 12, and 17

Even though the primary goal for this study is to identify the collaborative subnetworks of TFs due to the nature of the data we used, some data yielded from the analyses may contain biological meaningful information. For example, some CCGs may play a contributing role to regeneration. We counted the numbers of TFs in subnetworks 1, 12, or 17 each CCG belongs to, and the result is shown in Supplemental Table 8, where the CCGs are shown is three groups: (1) the CCGs that are not a TF, defined as non-TF CCGs. Due to the discrepancies in functions of subnetwork 1, 12, and 17, the non-TF CCGs that are usually not shared by the three subnetworks, especially between Subnetwork 1 and the other two subnetworks. Relatively speaking, subnetwork 12 and 17 shared more common non-TF CCGs than any of them with Subnetwork 1. Based on the gene ontology (Supplemental Tables 4, 5, and 6) and gene functional annotation, the CCGs of Subnetwork 1, are primarily involved seed and embryo development; the CCGs of Subnetwork 12 are primarily involved in organ, root, root cap, tissue development, meristem growth and development as well as shoot development, and the CCGs of Subnetwork 17 are primarily involved in root and leaf development, meristem development, and callus formation. The nonTF CCGs account for 88.6% of all CCGs of Subnetworks 1, 12, or 17; (2) The CCGs themselves are a TF but not the one in the Subnetwork 1, 12, and 17; only 2% CCGs of Subnetworks 1,12, and 17 are of this type. (3) The CCGs themselves are a TF in Subnetwork 1, 12, or 17 and belong to another TF in the three subnetworks. About 9.4% CCGs of Subnetworks 1,12, and 17 are of this type. Interested readers may look up Supplemental Table 8 for CCGs that may potentially contribute to the regeneration.

### Motif enrichment analysis

MotifLocator and the CIS-BP database were used to search for known TFBSs in the CCG’s promoter regions. As detailed in Table 2A–C, MotifLocator successfully identified known TFBSs for only a subset of key pluripotency regulatory genes. In these tables, columns 6, 7, and 8 provided information about the frequency of motifs in the CCGs, the proportions of CCGs containing each motif, and the statistical significance of each motif determined by the hypergeometric test, respectively. In Table 2A, a total of four motifs were identified for key pluripotency genes *LEC2* and *PGA37* of Subnetwork 1. Likewise, three motifs were found for PLT1 and ARF16 in Subnetwork 12 (Table 2B), while eight motifs were identified for LBD16, PLT3, and BRAVO in Subnetwork 17 (Table 2C). Furthermore, it's worth noting that the hypergeometric test only successfully enriched the motifs in the CCGs of Subnetwork 1 compared to genome-wide TFBS distribution.

**Table 2:**
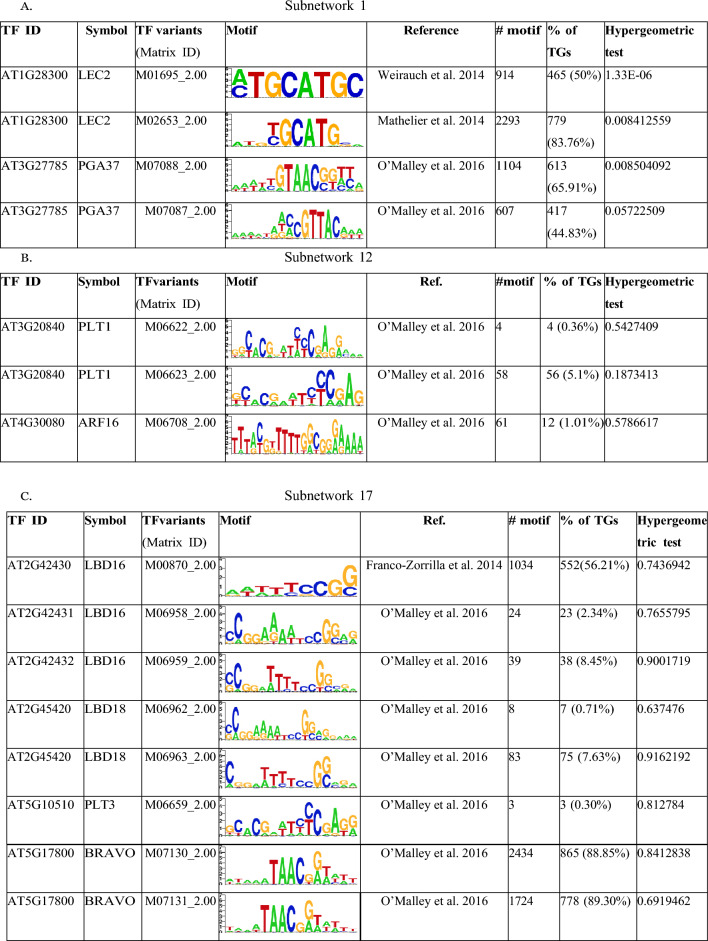
Transcription factor binding sites (TFBSs) searched for transcription factors (TFs) in the promoter regions of co-expressed and co-regulated genes (CCGs) of each TF in a collaborative subnetwork 1, 12, and 17

The number of occurrences for a particular motif, % of CCGs containing that motif in their promoter regions, and hypergeometric test against whole genome set are enlisted in the table. Column four represents the sequence logo of the motif using PWM

### Validation and ranking TFs in each collaborative subnetworks using TGMI algorithm

**Fig. 6:**
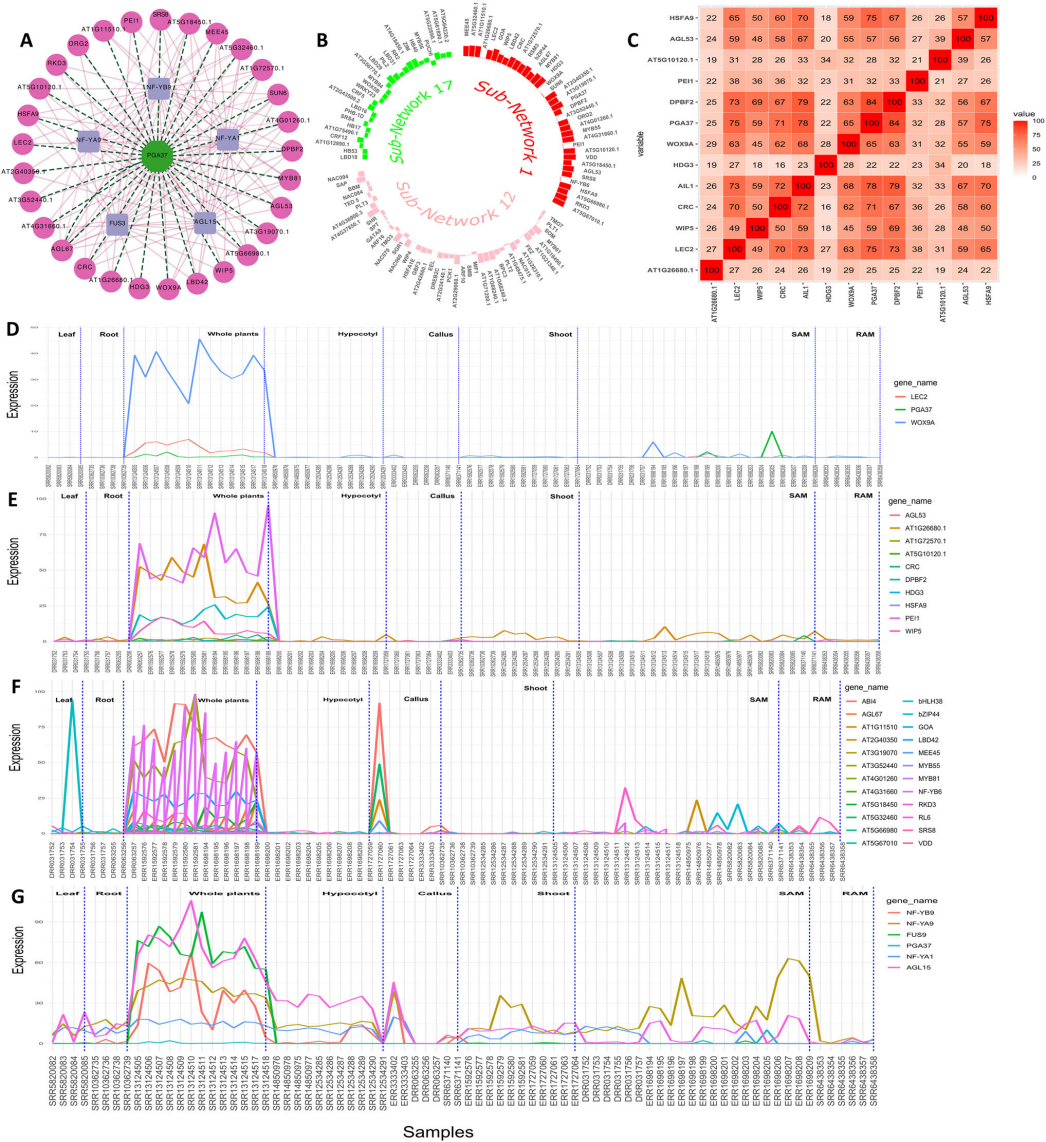
Comprehensive analysis and visualization of subnetwork 1 transcription factors (TFs) and networks related to embryogenesis and plant regeneration. **A** Subnetwork 1 TFs and their interfered genes resulting from the triple-gene mutual interaction (TGMI) analysis on this subnetwork. Pink circles depict TFs, light violet rectangles represent the genes involved in somatic embryogenesis and regeneration, and the green octagon represents a gene which acts as both a TF and a CCG. **B** Circular plot of Subnetworks 1, 12 and 17 considering Uniform Manifold Approximation and Projection (UMAP). The size and direction of the bar for each gene depict the value found from UMAP. **C** A sub-matrix of Shared Co-expression Connectivity Matrix (SCCM) values of the 13 most significant TFs in subnetwork 1. **D**–**G** Expression profiles of different sets of genes across 78 samples. **D** Three proven pluripotent TFs: *LEC2*, *PGA37*, and *WOX9A*. **E** Ten new potential TFs of interest. **F** The 24 remaining TFs in Subnetwork 1. **G** Six regulatory genes involved in somatic embryogenesis (GO:0010262) and regeneration (GO:0031099)

We used TGMI to evaluate each TF’s interference frequency on a specific biological process. These include TFs in Subnetwork 1 and the somatic embryogenesis (GO:0010262) and the regeneration (GO:0031099) that were enriched in CCGs of Subnetwork 1. For the TGMI analysis, we merged the biological process genes involved in both the somatic embryogenesis and regeneration, and then combined each TF in Subnetwork 1 with two biological process genes from the merged somatic embryogenesis and regeneration processes as a trio for evaluation. When all combinations of the possible trios were assessed, and the number of times each TF interfered with each biological gene present in various trios was calculated (Table 3) (Supplemental Table 9). CRC, AIL1, WIP5, WOX9A, LEC2, PGA37 and PEI1 were among this result. We also built a TF-TG gene network to represent their regulatory interrelationships (Fig. 6A). Column 1 indicates the ranking of all the TFs in Subnetwork 1 depending on their frequencies and MIM values.

**Table 3:** Summary of the sub-network 1 transcription factor interference frequencies on genes involved in somatic embryogenesis (GO:0010262) and the regeneration (GO:0031099) by Triple-Gene Mutual Information (TGMI) algorithm and the binding site motif analysis of sub-network 1 TFs in their co-expressed and co-regulated genes (CCGs)

Rank	TF Id	Symbol	Frequency	# Motif	References
1	AT1G69180.1	CRC	6	98	Bowman and Smyth (1999)
2	AT1G72570.1	AIL1	6	0	Nole-Wilson et al. (2005)
3	AT1G77950.1	AGL67	6	0	
4	AT2G26960.1	MYB81	6	75	
5	AT2G40220.1	SUN6	6	0	
6	AT3G44460.1	DPBF2	6	0	Bensmihen et al. (2005)
7	AT4G00260.1	MEE45	6	0	
8	AT4G01260.1	AT4G01260.1	6	0	
9	AT5G18450.1	AT5G18450.1	6	2	
10	AT5G32460.1	AT5G32460.1	6	0	
11	AT5G33210.2	SRS8	6	0	
12	AT5G66980.1	AT5G66980.1	6	0	
13	AT1G68510.1	LBD42	5	0	
14	AT5G27070.1	AGL53	5	0	Lehti-Shiu et al. (2005)
15	AT5G54070.1	HSFA9	5	0	Kotak et al. (2007)
16	AT1G51220.1	WIP5	4	2	Crawford et al. (2015)
17	AT2G33880.1	WOX9A	4	0	Breuninger et al. (2008)
18	AT4G31660.1	AT4G31660.1	4	0	
19	AT5G10120.1	AT5G10120.1	4	0	Cao et al. (2006)
20	AT5G66990.1	RKD3	4	0	
21	AT1G11510.1	AT1G11510.1	3	0	
22	AT1G26680.1	AT1G26680.1	3	0	Galla et al. (2015)
23	AT1G28300.1	LEC2	3	26	Wojcikowska et al. (2013)
24	AT2G40350.1	AT2G40350.1	3	0	
25	AT3G19070.1	AT3G19070.1	3	0	
26	AT3G27785.1	PGA37	3	11	
27	AT3G52440.1	AT3G52440.1	3	67	
28	AT3G56970.1	ORG2	3	0	
29	AT5G07500.1	PEI1	3	0	Hubberten et al. (2015)
30	AT2G32370.1	HDG3	2	0	Pignatta et al. (2018)

We also used TGMI to evaluate TFs in Subnetwork 12 on their interference frequencies with the biological process genes involved in embryo development (GO:0009790) (Supplemental Table 10), and TFs in Subnetwork 17 on their interference frequencies with embryo development (GO:0009790) and callus formation (GO:1990110) (Supplemental Tables 11 and 12, respectively). For Subnetwork 12, the TFs that interfered with the embryo development included TRO7, PLT1, PLT2 BBM, PLT3, and WIP4. For Subnetwork 17, the TFs that interfered with the embryo development included LBD31, PLT3, LBD16, LBD18, MYB56, WOX5B, etc., while the TFs that interfered with the callus formation included MYB56, LBD16, PLT3, WOX5B, and LBD29. Column 4 in Table 3 shows the frequency of each TF that interfered with the biological process genes of somatic embryogenesis and regeneration, while column 5 represents the number of motifs found in the proximal promoters of the CCGs involved in somatic embryogenesis and regeneration. We then performed an extensive literature search to determine the biological relevance of our high-ranked TFs. *WOX9A*, *LEC2*, and *PGA37*, were highly-ranked in the TGMI analysis, and they are also proven pluripotent genes. However, some other top-ranked genes, such as *CRC*, *AIL1*, *DPBF2*, *AGL53*, *HSFA9*, *WIP5*, *EIL4*, *AT1G26680*, *PEI1*, *HDF3*, etc., may also be directly or indirectly regulate regeneration, we will discuss some of them in Discussion and some additional candidate genes (CRC, DPBF2, NF-YB6, EIL4, HDG3, HSFA9, SMB, FEZ, NAC015, NAC070, etc.) in Supplemental File 1.

## Discussion

Plants possess remarkable regenerative capabilities, especially in tissues exhibiting pluripotency, multipotency, or those affected by injuries. For example, SAMs generate leaves and stems (Mathew and Prasad 2021), and vascular cambium cylinders beneath tree barks divide inwardly to produce wood, and outwardly to produce the tree barks (Wang et al. 2021). In addition, somatic cells can be cultured in a growth medium containing balanced plant hormones (auxins and cytokinins), leading to cell proliferation and ultimately regaining totipotency. This process can result in the development of embryos, and subsequently, entire plants (Fehér 2015). When plants are wounded, some cells in the wounded sites undergo de-differentiation, giving rise to pluripotent and multipotent cells that can generate new tissues or organs. For instance, stem cuttings of poplar trees can produce the new roots (Wang et al. 2022). Network analyses have indicated that wound- and/or hormone-invoked signals exhibit extensive cross-talk and regulate many common reprogramming-associated genes via multilayered regulatory cascades (Ikeuchi et al. 2018). Therefore, we had included 145 regulatory genes involved in pluripotency and multipotency maintenance, and regeneration, as shown in Supplemental Table 1. These 145 genes play intricate roles in different stages of regeneration by regulating the expression of their target genes. *WUS*, for example, promotes stem cell identity in SAM for mass production and proliferation in differentiation phase, and *CLAVATA* restricts WUS expression to manage the stem cell population (Mayer et al. 1998). In a similar manner, *WIND1* controls de-differentiation in order to facilitate the production of calli in response to a wound (Iwase et al. 2011). In the re-differentiation phase, *PLT1* and *PLT2* genes, both encoding for putative AP2 class TFs, are required for the specification of the quiescent center and the activity of stem cells. However, it is a fact that some key TFs with direct or indirect involvement in the regeneration process remain unidentified. To address the void, we developed a bioinformatics-based pipeline to identify important TFs crucial for regulating the biological processes involved in regeneration.

Our study on regeneration commenced with an in-depth exploration of the mechanisms involved in the regeneration process of *A. thaliana*. This allowed us to gain insights into the various tissues that may have undergone transitions related to pluripotency and multipotency. Based on the knowledge, we collected the metadata of *A. thaliana* from NCBI database, and carefully curated and selected a subset of 78 samples that specifically pertain to calli, meristematic tissues (SAM and RAM), and somatic cells induced from callus induced differentiated somatic cells (leaf, root, shoot, hypocotyl, and whole explants) (Supplemental Table 2). Next, the samples were preprocessed by our pipelines, which includes acquiring FASTQ files, conducting quality control and read trimming, and aligning the processed clean reads to the *A. thaliana* reference genome, as well as quantifying transcripts to gain gene expression values in the form of FPKM. The construction of a collaborative network, followed by its decomposition using the Triple-Link algorithm led to the generation of 280 non-overlapping subnetworks. CollaborativeNet prioritizeed subnetworks based on coordination strengths, ranking those with high coordination strength first. The algorithm's preference for TF pairs demonstrating the highest coordination strength within SCCM for a subnetwork decomposition led to this prioritization. Typically, the top 100 subnetworks are more biologically meaningful, while those beyond 100 tend to be smaller, and often containing just a few TFs as shown in boxplot (Supplemental Fig. 2). Through the alignment of known pluripotent TFs (Supplemental Table 1) with subnetworks TFs and employing gene annotation-based selection, we narrowed down the initially identified subnetworks to just nine (Fig. 1). The application of CoSE to the SCCM resulted in almost the same nine subnetworks (Fig. 2). PCA-based analysis identified four subnetworks: Subnetworks 1, 12, 17, and 74, which exhibited the most promising roles in the regeneration process when compared to the others (Fig. 3). Finally, we performed GO analysis using the 980, 1116, 997, and 559 CCGs associated with these four subnetworks in the above order, respectively. The result, as shown in Fig. 5, indicated that biological processes related to regeneration and somatic embryogenesis were significantly enriched in Subnetwork 1, as indicated by adjusted *P*-values of 0.02 and 0.007, respectively. Furthermore, these two biological processes have the highest fold-enrichment values of 8.95 and 11.7 respectively. Additionally, other GO terms in the CCGs of subnetwork 1 include ‘seed maturation’, ‘seed development’, ‘multicellular organism reproduction’, ‘multicellular organismal reproductive process’, ‘developmental maturation’, ‘embryo development ending in seed dormancy’, ‘embryo development’, ‘reproductive structure development’, ‘reproductive system development’, ‘post-embryonic development’, and functions of regeneration and somatic embryogenesis (Fig. 4A, B and Supplemental Table 4). To further explore the characteristics of these subnetworks, we employed the Uniform Manifold Approximation and Projection (UMAP), a dimensional reduction technique used for visualizing high-dimensional data. As depicted in Fig. 6B, it was discovered that Subnetwork 1 exhibited predominantly positive directionality. Subnetworks 12 and 17, on the other hand, exhibit a mix of positive and negative values. This suggests that the TFs within Subnetwork 1 consistently exhibit positive values.

**Fig. 7:**
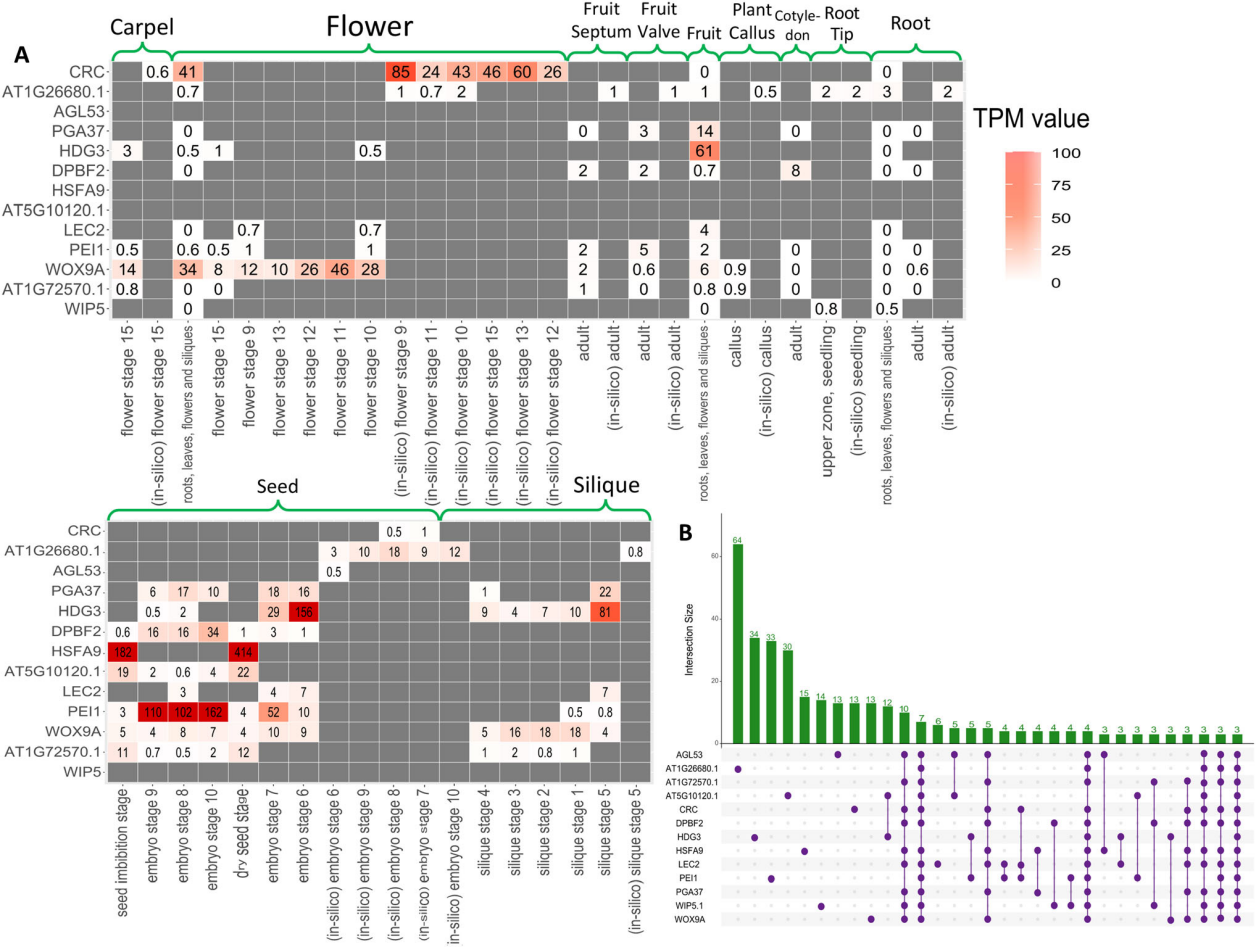
**A** Heatmap of various tissues’ gene expression values in transcripts per million (TPM) in various developmental stages. The data was collected from Expression Atlas. **B** UpSet plot of the 13 TFs illustrating the shared number of CCGs

TFs exert control over the expression of their target genes by binding to the promoter regions, thus influencing the up- or down-regulation of various biological processes. To identify the roles of the TFs we identified, we investigated the presence of transcription factor binding sites (TFBS) within their associated CCGs. Our analysis revealed an enrichment of the binding motifs of the TFs from Subnetwork 1 in their CCGs, as confirmed by the hypergeometric test (Table 2A). However, we were unable to detect enriched motifs of Subnetworks 12 and 17’s TFs in their CCGs (Table 2B, C). To refine and rank TFs in Subnetwork 1, we utilized TGMI to infer the interference frequencies of TFs with respect to the genes associated with somatic embryogenesis and regeneration. Here, the gene expression data of 39 TFs in Subnetwork 1 and six biological process genes from regeneration and somatic embryogenesis were used for TGMI analysis. TGMI ranked the TFs based on their interference frequency of with the six biological process genes (Table 3; Fig. 6A). Furthermore, we performed motif enrichment analysis for these ranked TFs as presented in Table 3. Finally, we identified the 13 most significant TFs of interest from Subnetwork 1, which may be directly or indirectly associated with the regeneration process. These includes *CRC*, *AIL1*, *DPBF2*, *AGL53*, *HSFA9*, *WIP5*, *WOX9A*, *AT5G10120.1/EIL4*, *AT1G26680.1*, *LEC2*, *PGA37*, *PEI1*, and *HDG3*. Notably, among these 13 TFs, LEC2, PGA37, and WOX9A have already been linked to regeneration in the current literature (Fig. 2). To further validate these TFs, we used an online literature-based database called Expression Atlas (Papatheodorou et al. 2018), considering their expression data and tissue specificity (Fig. 7A). Even though the dataset used by this tool comprises a small fraction of data originating from cells or tissues with pluripotency and multipotency, these 13 TFs, and especially WOX9A, LEC2, PGA37, PEI1, AIL1, and WIP5, exhibited common patterns and connections. This observation aligns with a sub-network centered around these 13 TFs, as displayed in Figs. 6C and 7B.

The regulatory roles of *WOX9A*, *LEC2* and *PGA37* in regeneration and/or embryogenesis have been previously been demonstrated by both in vivo and in vitro level experiments (Long et al. 2023; Ma et al. 2019; Wang et al. 2009; Wojcikowska et al. 2013), and also by our results, as shown in Tables 1 and 2A. *WOX9A* plays a direct and significant role in embryonic patterning and stem cell maintenance in plants, specifically in influencing the growth of both apical and basal cell lineages in *A. thaliana*’s whole plants (also highly expressed in whole plants as shown in Fig. 6D). This gene specifically regulates the growth of the SAM in part by positively affecting the expression of another gene, *WUS*, which plays a major role in maintaining the stem cells within SAM (Ma et al. 2019), WUSCHEL acts as an auxin response rheostat to maintain apical stem cells in *Arabidopsis*. *FWA* is a gene known to repress the transcription of *WOX9* by binding to its promoter regions. When *FWA* is overexpressed, it inhibits shoot regeneration, at least in part, by suppressing the transcription of *WOX9*. WOX9 has recently been shown to express constitutively in somatic embryogenesis in *Liriodendron* (Long et al. 2023). Thereby, *FWA* controls the shoot regeneration (Dai et al. 2021). *LEC2* is crucial for embryogenesis and seed development. It controls late embryogenesis and can induce somatic embryos from somatic cells in tissue culture spontaneously (Wojcikowska et al. 2013). In conjunction with *LEC1*, *FUS3*, and ABI3, *LEC2* stands as one of the four key regulators of embryonic identity. It also modulates the expression of FUS3 and ABI3, contributing to the establishment of a complex network that regulates seed maturation (Fig. 7A). Wang et al. conducted a study characterizing the roles of PLANT GROWTH ACTIVATOR37 (*PGA37*)/*MYB118* in the regulation of somatic embryogenesis and the transition from vegetative to embryonic states (Wang et al. 2009).

*AIL1*, *CRC*, *WIP5*, and *PEI1* are ranked in the analysis with 6, 6, 4, and 3 frequencies in interfering with the genes involved in somatic embryogenesis and regeneration, respectively, as shown in Table 3. Therefore, they may have great potential to regulate regeneration. *AIL1*, *WIP5*, and *PEI1* are actually supported by existing literature. The specific functions of WIP5’s in *A. thaliana* have not been determined; however, Wound-Induced Proteins (WIPs) trigger damage repair and regeneration. During the globular stage of embryonic development, *WIP5*, along with *NTT* and *WIP4*, is expressed in the hypophysis cells. This expression continues as the hypophysis produces the apical and basal daughter cells, redundantly facilitating the initiation of the root meristem (Crawford et al. 2015). *PEI1*, an embryo-specific zinc finger protein, plays a direct role in embryogenesis regulation and required for heart-stage embryo formation in *Arabidopsis* (Hubberten et al. 2015). *AIL1* /AT1G72570 is a AP2/ERF TFs family member, and a member of AP2 subgroup called AINTEGUMENTA-like group, which includes *BBM*, *PLT1-2*, *AILs*, and *ANT* in *Arabidopsis*. This subgroup is important for identity and maintenance of stem cells in RAM as well as development of embryos, leaves, and flowers. For example, BBM and AIL proteins interact with multiple members of HDG proteins, and overexpression of several HDG proteins causes the arrest of shoots/roots and meristematic tissues (Horstman et al. 2015). *AIL1* has been implicated in the regulation of cell proliferation and differentiation, contributing to the development of leaf- and flower-like organs (Nole-Wilson et al. 2005). We also discussed more potential TFs for pluripotency, such as CRC, EIL4, AT1G26680, HDG3, and HSFA9 (Supplemental File 1).

*AGL53*, a MADS-box transcription factor, plays a crucial role in controlling the growth of *A. thaliana* embryos and their potential to develop into whole plants (Lehti-Shiu et al. 2005). AGL53 was also expressed in whole plants regions as denoted in Fig. 6E. Several other transcription factors, including bZIP67/DPBF2, EIL4, HSFA9, and AT1G26680, are known to regulate the seed development process (GO:0048316) (Bies-Etheve et al. 2008; Camehl et al. 2010; Galla et al. 2015; Kim et al. 2022; Kotak et al. 2007). As shown in Fig. 4, embryo-development-ending-in-seed (GO:0009793) is a part of seed development process; and somatic embryogenesis is a type of GO:0009793. Moreover, it is possible that the remaining 24 TFs in Subnetwork 1 may have direct or indirect roles in regeneration. Most of them show higher expression in whole plants regions; however, *RL6*, *GOA*, and AT1G11510 show considerable expression in SAM and RAM samples. Particularly, *ABI4*, AT5G67010, AT1G11510, and *LBD42* showed distinct expression in callus samples as shown in Fig. 6F. Our procedure and tools have the potential to unveil known pluripotent TFs as well as predict strong candidate TFs for regeneration, such as *WIP5*, *PEI1*, and *AIL1*/*AT1G72570*.

TGMI was also applied to identify the TFs in Subnetworks 12 and 17 that may potentially regulate embryo development, and callus formation (Supplemental Table 10, 11 and 12). In Subnetwork 12, the TFs that interfered with the embryo development included TRO7, PLT1, PLT2 BBM, PLT3, and WIP4. For Subnetwork 17, the TFs that interfered with the embryo development included LBD31, PLT3, LBD16, LBD18, MYB56, and WOX5B, and the TFs that interfered with the callus formation included MYB56, LBD16, PLT3, WOX5B and LBD29. Many of these are known TFs that regulate the regeneration-related biological processes. For example, PLT1-3 and BBM are involved in early embryo development (ten Hove et al. 2015), LBD16 (Xu et al. 2018b), and LBD29 (Xu et al. 2018a) are involved in callus formation, indicating the high accuracy of the CollaborativeNet and TGMI algorithm. Which are consistent with a wealth of evidence we showed in our previous publications (Gunasekara et al. 2018; Ji et al. 2017; Nie et al. 2011). One of the main strengths of our study lies in the integration of biological evaluation throughout each stage of the procedure. The transcription factors (TFs) we've identified either align closely with existing knowledge or are supported by multiple lines of evidence and gene annotation. We believe that our methods hold great promise for investigating a wide range of other biological processes. However, it's essential to acknowledge a major limitation in our pipeline: it relies exclusively on computational approaches within an in silico environment. Therefore, further research is imperative, particularly at the in vivo and in vitro levels, to validate and substantiate the roles of the identified TFs. Additionally, the availability of data presents a significant hurdle in achieving a comprehensive assessment and fulfilling the objectives of our study. The reason for lack of bulk RNA data for such analysis in any plant species is primarily due to the difficulty in acquisition of the meristematic tissues for RNA-seq. These tissues are typically small, hard to dissect and insufficient for regular RNA-seq experiments. However, with the emergence of single-cell RNA-seq, we anticipate that more data will become accessible in future (Liao and Wang 2023).

## Conclusion

The collaborative network is one of the most efficient approaches to identify TFs that collaboratively regulate a biological process or a complex trait. The implementation of this method to transcriptomic data from *Arabidopsis* led to the identification of nine collaborative subnetworks. Three of them, Subnetworks 1, 12, and 17, were functionally associated with regeneration. GO enrichment analysis, TFBS enrichment analysis, and the TGMI algorithm were used to evaluate these three subnetworks and led to the identification of TFs that regulate regeneration and related processes. We found that subnetwork 1 mainly regulates regeneration and somatic embryogenesis. It contains 13 crucial TFs that interfered with the regeneration process. WOX9A, LEC2, and PGA37 are well-established, important regulators in regeneration and embryogenesis, and their rediscovery here manifests the precision and viability of our approach. LEC2 is essential for embryogenesis and seed formation, while WOX9A maintains stem cells and embryonic patterning. Another notable outcome is that WIP5, PEI1, and AIL1 may play regulatory functions in regeneration. In addition, our study found known regulatory genes in subnetworks 12 and 17 that are evidenced to regulate embryo growth and callus formation, demonstrating that the algorithms used were effective and reliable. The rigorous biological examination at each step made the identified TFs congruent with existing understanding and knowledgebase. This study represents a major step in understanding how transcriptional regulation controls plant regeneration and embryogenesis. The TFs and the networks identified can help elucidate the regulatory mechanisms underlying plant regeneration and lay the basis for future research and prospective advances in plant biology.

### Supplementary Information

Below is the link to the electronic supplementary material.

Supplementary file1 (DOCX 44 kb)

Supplementary file2 (DOCX 410 kb)

Supplementary file3 (DOCX 253 kb)

Supplementary file4 (DOCX 43 kb)

Supplementary file5 (XLSX 14 kb)

Supplementary file6 (XLSX 340 kb)

Supplementary file7 (XLSX 12 kb)

Supplementary file8 (XLSX 13 kb)

Supplementary file9 (XLSX 13 kb)

Supplementary file10 (XLSX 23 kb)

Supplementary file11 (XLSX 132 kb)

Supplementary file12 (XLSX 18 kb)

Supplementary file13 (XLSX 19 kb)

Supplementary file14 (XLSX 16 kb)

Supplementary file15 (XLSX 16 kb)

## Data Availability

The datasets analyzed during the current study are available in the NCBI repository, [https://www.ncbi.nlm.nih.gov/sra]. CollaborativeNet (originally TF-Cluster) software is available at Github (https://github.com/hwei0805/TF_CollaborativeNet).
