# Supplementary material for: Regulation of regeneration in Arabidopsis thaliana
Source: aBIOTECH. 2023 Nov 22;4(4):332–51. doi: 10.1007/s42994-023-00121-9 (PMC10721781; doi:10.1007/s42994-023-00121-9)
Supplement: Supplementary file 1 — Supplementary file1 (DOCX 44 kb) [file 42994_2023_121_MOESM1_ESM.docx]

Regulation of Regeneration in Arabidopsis thaliana

Md Khairul Islam^a,d^, Sai Teja Mummadi^b^, Sanzhen Liu^c^, Hairong Wei^a,b,d^

*^a^Computational Science and Engineering Program, Michigan Technological University, Houghton, 49931, Michigan, United States of America*

*^b^Computer Science, Michigan Technological University, Houghton, 49931, Michigan, United States of America*

*^c^Department of Plant Pathology, Kansas State University, Manhattan, 66506, Kansas, United States of America*

*^d^College of Forest Resources and Environmental Science, Michigan Technological University, Houghton, 49931, Michigan, United States of America*

**More discussion of the potential regulators of regeneration and embryonic regeneration**

In addition to the known regulators (e.g., LEC2, WOX9A. PGA37) in Subnetwork 1, there are several other regulators that may function in regeneration. The YABBY Family’s CRABS CLAW (CRC), ranked at the top in the TGMI analysis (Table 4) is a transcription factor that involved in floral meristem termination, gynoecium differentiation, and ovule initiation ^1^. CRC is a direct transcriptional repressor of plasma membrane-localized TRN2 that modulates auxin homeostasis ^2^. CRC can be induced by FUS3 and ABI5 in vegetative tissues and seed development ^3^. DPBF2, which encodes the seed-specific DC3 PROMOTER-BINDING FACTOR2 was upregulated by LEC2, which, together with ABI3 and FUSCA3, play key roles in the control of seed maturation in cooperation with LEC1 ^4^. NF-YB6, a LEC1-like gene, is essential in seed embryo development ^5^. The role of EIL4 in seed development has not been studied as much as that of its family member EIN3, EIL4 likely plays a role in seed development processes ^6^. AT1G26680 is an Arabidopsis gene that encodes a member of the B3 family transcription factor and has a role in seed development ^7^. But some low expression levels were found randomly in different stages of tissue development, as shown in Figure-7A, even though not enough information was found in the literature. When the HDG3 gene is methylated, it causes the gene to be activated and imprinted ^8^. Imprinted genes play an important role in the creation and expansion of the seed endosperm, a tissue that feeds the growing embryo ^9^. HSFA9, regulated by a seed-specific transcription factor, ABI3, is exclusively expressed in the late stages of seed development. It also controls the LEA proteins, which are essential for the plant embryo’s survival under dehydration and other stressors^10,11^. In Figure 7, HSFA9 was highly expressed in dry seed stage and seed imbibition stage that enable the seeds to survive in their dry, dormant state and prepare them for germination. HSFA9 does not directly trigger the process of regeneration while being expressed during late seed development. Finally, bZIP44, a transcription factor, affects seed germination^12^. It’s possible that these genes may have an indirect role in regeneration as embryo development ending in seed dormancy (GO:0009793), which is a sub-process of embryo development (GO:0009790), while somatic embryogenesis (GO:0010262) is a sub-process of GO:0009793 (Figure 4A). In addition to somatic embryogenesis, tissue, shoot and root regeneration are a subprocess of regeneration (GO:0031099). ORG3 regulated by GRF9 restricts cell proliferation in leaf primordia ^13^.

In addition to some known genes (e.g., BBM, PLT1, 2, 3) in Subnetwork 12, there are several genes that may be involved in stem cell growth and maintenance. These include SMB, FEZ, NAC015 (BRN1), and NAC070 (BRN2). FEZ promotes periclinal, root cap-forming cell divisions while SMB represses stem cell-like divisions in root cap daughter cells via negatively regulating FEZ activity ^14^. BRN1 and 2 are expressed in root cap and act redundantly in root cap maturation ^15^. TMO7 functions in the founder cells of the root cap and RNA suppression experiments implicated its function in embryonic root development ^16^. ARF16 regulates auxin signaling pathway that play a key role in LEC2-mediated somatic embryogenesis ^17,18^

Subnetwork 17 contains multiple known genes (LBD29, 16, 18, 31, and PLT3) involved in callus formation. In addition to that, CRF5 functions redundantly to regulate the development of embryos, cotyledons and leaves. Genes encoding homeobox transcription factors are mis-expressed in the *crf5* mutant, including *WOX9* that is required for root and shoot apical meristem maintenance ^19^. There is also a CRF12 in Subnetwork 17, however, its functions have not been characterized.

**References**

1. Orashakova, S., Lange, M., Lange, S., Wege, S., and Becker, A. (2009). The CRABS CLAW ortholog from California poppy (Eschscholzia californica, Papaveraceae), EcCRC, is involved in floral meristem termination, gynoecium differentiation and ovule initiation. Plant J *58*, 682-693. 10.1111/j.1365-313X.2009.03807.x.

2. Yamaguchi, N., Huang, J., Xu, Y., Tanoi, K., and Ito, T. (2017). Fine-tuning of auxin homeostasis governs the transition from floral stem cell maintenance to gynoecium formation. Nat Commun *8*, 1125. 10.1038/s41467-017-01252-6.

3. Kagaya, Y., Okuda, R., Ban, A., Toyoshima, R., Tsutsumida, K., Usui, H., Yamamoto, A., and Hattori, T. (2005). Indirect ABA-dependent regulation of seed storage protein genes by FUSCA3 transcription factor in Arabidopsis. Plant Cell Physiol *46*, 300-311. 10.1093/pcp/pci031.

4. Vicente-Carbajosa, J., and Carbonero, P. (2005). Seed maturation: developing an intrusive phase to accomplish a quiescent state. Int J Dev Biol *49*, 645-651. 10.1387/ijdb.052046jc.

5. Kwong, R.W., Bui, A.Q., Lee, H., Kwong, L.W., Fischer, R.L., Goldberg, R.B., and Harada, J.J. (2003). LEAFY COTYLEDON1-LIKE defines a class of regulators essential for embryo development. Plant Cell *15*, 5-18. 10.1105/tpc.006973.

6. Camehl, I., Sherameti, I., Venus, Y., Bethke, G., Varma, A., Lee, J., and Oelmuller, R. (2010). Ethylene signalling and ethylene-targeted transcription factors are required to balance beneficial and nonbeneficial traits in the symbiosis between the endophytic fungus Piriformospora indica and Arabidopsis thaliana. New Phytol *185*, 1062-1073. 10.1111/j.1469-8137.2009.03149.x.

7. Galla, G., Vogel, H., Sharbel, T.F., and Barcaccia, G. (2015). De novo sequencing of the Hypericum perforatum L. flower transcriptome to identify potential genes that are related to plant reproduction sensu lato. BMC Genomics *16*, 254. 10.1186/s12864-015-1439-y.

8. Pignatta, D., Novitzky, K., Satyaki, P.R.V., and Gehring, M. (2018). A variably imprinted epiallele impacts seed development. PLoS Genet *14*, e1007469. 10.1371/journal.pgen.1007469.

9. Batista, R.A., Figueiredo, D.D., Santos-Gonzalez, J., and Kohler, C. (2019). Auxin regulates endosperm cellularization in Arabidopsis. Genes Dev *33*, 466-476. 10.1101/gad.316554.118.

10. Kotak, S., Vierling, E., Baumlein, H., and von Koskull-Doring, P. (2007). A novel transcriptional cascade regulating expression of heat stress proteins during seed development of Arabidopsis. Plant Cell *19*, 182-195. 10.1105/tpc.106.048165.

11. Bies-Etheve, N., Gaubier-Comella, P., Debures, A., Lasserre, E., Jobet, E., Raynal, M., Cooke, R., and Delseny, M. (2008). Inventory, evolution and expression profiling diversity of the LEA (late embryogenesis abundant) protein gene family in Arabidopsis thaliana. Plant Mol Biol *67*, 107-124. 10.1007/s11103-008-9304-x.

12. Iglesias-Fernandez, R., Barrero-Sicilia, C., Carrillo-Barral, N., Onate-Sanchez, L., and Carbonero, P. (2013). Arabidopsis thaliana bZIP44: a transcription factor affecting seed germination and expression of the mannanase-encoding gene AtMAN7. Plant J *74*, 767-780. 10.1111/tpj.12162.

13. Omidbakhshfard, M.A., Fujikura, U., Olas, J.J., Xue, G.P., Balazadeh, S., and Mueller-Roeber, B. (2018). GROWTH-REGULATING FACTOR 9 negatively regulates arabidopsis leaf growth by controlling ORG3 and restricting cell proliferation in leaf primordia. PLoS Genet *14*, e1007484. 10.1371/journal.pgen.1007484.

14. Willemsen, V., Bauch, M., Bennett, T., Campilho, A., Wolkenfelt, H., Xu, J., Haseloff, J., and Scheres, B. (2008). The NAC domain transcription factors FEZ and SOMBRERO control the orientation of cell division plane in Arabidopsis root stem cells. Dev Cell *15*, 913-922. 10.1016/j.devcel.2008.09.019.

15. Bennett, T., van den Toorn, A., Sanchez-Perez, G.F., Campilho, A., Willemsen, V., Snel, B., and Scheres, B. (2010). SOMBRERO, BEARSKIN1, and BEARSKIN2 regulate root cap maturation in Arabidopsis. Plant Cell *22*, 640-654. 10.1105/tpc.109.072272.

16. Lu, K.J., De Rybel, B., van Mourik, H., and Weijers, D. (2018). Regulation of intercellular TARGET OF MONOPTEROS 7 protein transport in the Arabidopsis root. Development *145*. 10.1242/dev.152892.

17. Wojcik, A.M., Nodine, M.D., and Gaj, M.D. (2017). miR160 and miR166/165 Contribute to the LEC2-Mediated Auxin Response Involved in the Somatic Embryogenesis Induction in Arabidopsis. Front Plant Sci *8*, 2024. 10.3389/fpls.2017.02024.

18. Wojcikowska, B., and Gaj, M.D. (2017). Expression profiling of AUXIN RESPONSE FACTOR genes during somatic embryogenesis induction in Arabidopsis. Plant Cell Rep *36*, 843-858. 10.1007/s00299-017-2114-3.

19. Raines, T., Shanks, C., Cheng, C.Y., McPherson, D., Argueso, C.T., Kim, H.J., Franco-Zorrilla, J.M., Lopez-Vidriero, I., Solano, R., Vankova, R., et al. (2016). The cytokinin response factors modulate root and shoot growth and promote leaf senescence in Arabidopsis. Plant J *85*, 134-147. 10.1111/tpj.13097.
