# Supplementary material for: Regulation of regeneration in Arabidopsis thaliana
Source: aBIOTECH. 2023 Nov 22;4(4):332–51. doi: 10.1007/s42994-023-00121-9 (PMC10721781; doi:10.1007/s42994-023-00121-9)

**Supplemental Figure 1**: The heatmap shows the gene expression values across 78 samples for the 145 pluripotent genes included in our analysis.


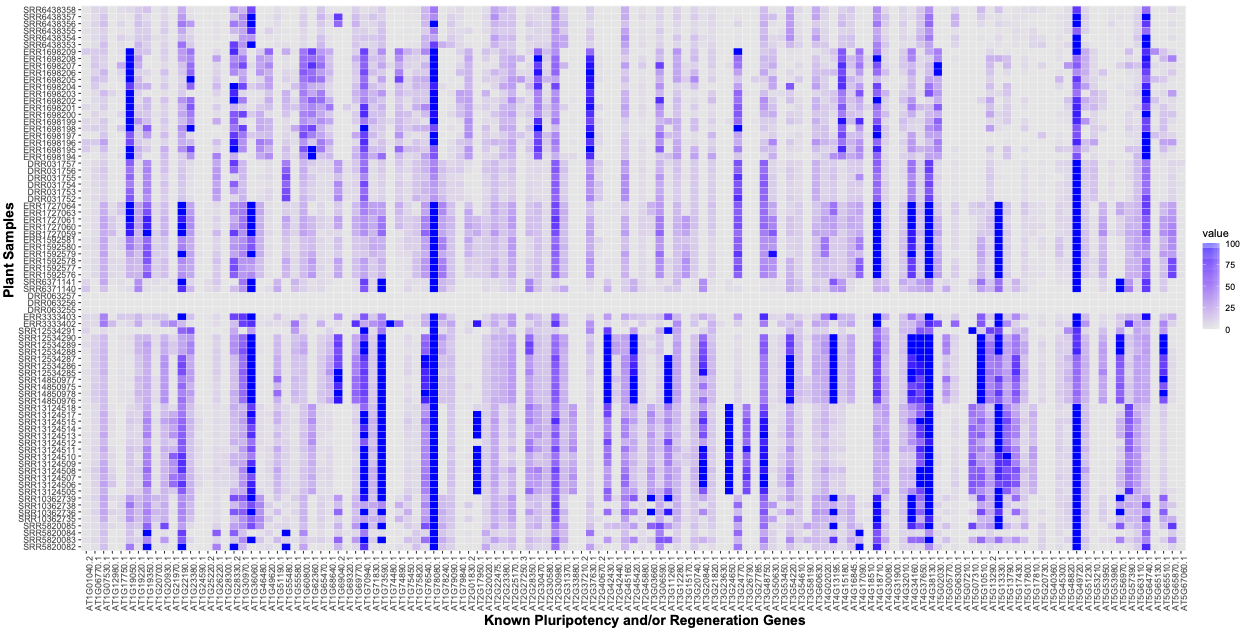

Supplement: Supplementary file 2 — Supplementary file2 (DOCX 410 kb) [file 42994_2023_121_MOESM2_ESM.docx]
