# Supplementary material for: Regulation of regeneration in Arabidopsis thaliana
Source: aBIOTECH. 2023 Nov 22;4(4):332–51. doi: 10.1007/s42994-023-00121-9 (PMC10721781; doi:10.1007/s42994-023-00121-9)
Supplement: Supplementary file 3 — Supplementary file3 (DOCX 253 kb) [file 42994_2023_121_MOESM3_ESM.docx]

**Supplemental Figure 2**: The boxplot represents the distribution of the number of common CCGs between all paired Transcription Factors (TFs) within a subnetwork for the first 150 subnetworks.
