# Supplementary material for: Regulation of regeneration in Arabidopsis thaliana
Source: aBIOTECH. 2023 Nov 22;4(4):332–51. doi: 10.1007/s42994-023-00121-9 (PMC10721781; doi:10.1007/s42994-023-00121-9)
Supplement: Supplementary file 4 — Supplementary file4 (DOCX 43 kb) [file 42994_2023_121_MOESM4_ESM.docx]

**Supplementary Table 1** Transcription Regulator (TFs) and non-TFs that are responsible for regeneration in Arabidopsis Thaliana. All the TFs and non-TFs are collected from previous literature-based proof.

1. TF Genes

| **No** | **TF Name** | **TF ID** | **Organism Part** | **Reference** |
| --- | --- | --- | --- | --- |
| TF 1 | BBM | AT5G17430 | RAM | (Horstman et al. 2017) |
| TF 2 | WOX5 | AT3G11260 | RAM | (Sarkar et al. 2007) |
| TF 3 | ESR1 | AT1G12980 | Root | (Banno et al. 2001) |
| TF 4 | LBD16 | AT2G42430 | lateral root | (Xu and Huang 2014) |
| TF 5 | LBD17 | AT2G42440 | lateral root | (Xu and Huang 2014) |
| TF 6 | LBD18 | AT2G45420 | lateral root | (Xu and Huang 2014) |
| TF 7 | LBD29 | AT3G58190 | lateral root | (Xu and Huang 2014) |
| TF 8 | CUC2 | AT5G53950 | SAM | (Xu and Huang 2014) |
| TF 9 | WUS | AT2G17950 | Shoot Stem cell | (Du 2020) |
| TF 10 | STM | AT1G62360 | Shoot Stem cell | (Du 2020) |
| TF 11 | CUC1 | AT3G15170 | SAM | (Aida et al. 2020) |
| TF 12 | PAN | AT1G68640 | SAM | (Aida et al. 2020) |
| TF 13 | BLR | AT5G02030 | SAM | (Aida et al. 2020) |
| TF 14 | LAS | AT1G55580 | SAM | (Aida et al. 2020) |
| TF 15 | KNAT6 | AT1G23380 | SAM | (Aida et al. 2020) |
| TF 16 | ESR2 | AT1G24590 | root explants | (Matsuo et al. 2011) |
| TF 17 | LEC1 | AT1G21970 | Shoot | (Tao et al. 2019) |
| TF 18 | LEC2 | AT1G28300 | Shoot | (Tao et al. 2019) |
| TF 19 | FUS3 | AT3G26790 | Shoot | (Tao et al. 2019) |
| TF 20 | ABI3 | AT3G24650 | — | (Xu and Huang 2014) |
| TF 21 | AGL15 | AT5G13790 | Shoot | (Xu and Huang 2014) |
| TF 22 | PGA37 | AT3G27785 | — | (Wang et al. 2009) |
| TF 23 | MYB115 | AT5G40360 | — | (Xu and Huang 2014) |
| TF 24 | EMK | AT5G57390 | — | (Xu and Huang 2014) |
| TF 25 | VAL1 | 2G30470 | — | (Xu and Huang 2014) |
| TF 26 | VAL2 | AT4G32010 | — | (Xu and Huang 2014) |
| TF 27 | WIND1 | AT1G78080 | — | (Xu and Huang 2014) |
| TF 28 | WOX9 | AT2G33880 | RAM | (Gaillochet and Lohmann 2015) |
| TF 29 | WOX14 | AT1G20700 | RAM | (Wang et al. 2022) |
| TF 30 | WOX7 | AT5G05770 | RAM | (Wang et al. 2022) |
| TF 31 | CRN | AT5G13290 | SAM | (Somssich et al. 2016) |
| TF 32 | ERF115 | AT5G07310 | ROOT | (Gaillochet and Lohmann 2015) |
| TF 33 | ARF16 | AT4G30080 | — | (Gaillochet and Lohmann 2015) |
| TF 34 | ARF10 | AT2G28350 | — | (Gaillochet and Lohmann 2015) |
| TF 35 | HAM2 | AT3G60630 | RAM | (Zhou et al. 2015) |
| TF 36 | CLF | AT2G23380 | Root | (Lee and Seo 2018) |
| TF 37 | AS1 | AT2G37630 | SAM | (Iwakawa et al. 2020) |
| TF 38 | AS2 | AT1G07530 | SAM | (Takahashi et al. 2013) |
| TF 39 | WOX4 | AT1G46480 | — | (Shimadzu et al. 2023) |
| TF 40 | PLT4 | AT5G17430 | ROOT | (Shim et al. 2020) |
| TF 41 | AtbZIP59 | AT2G31370 | — | (Sugiyama 2018) |
| TF 42 | HB52 | AT5G53980 | — | (Cheng et al. 2015) |
| TF 43 | RAP2.6L | AT5G13330 | — | (Ikeuchi et al. 2013) |
| TF 44 | HSL1 | AT4G32010 | LEAF | (Fletcher 2020) |
| TF 45 | WIND2 | AT1G22190 | callus | (Shin and Seo 2018) |
| TF 46 | SPCH | AT5G53210 | LEAF | (Fletcher 2020) |
| TF 47 | WIND1 | AT1G78080 | callus | (Shin and Seo 2018) |
| TF 48 | SUF4 | AT1G30970 | — | (Liu et al. 2015) |
| TF 49 | AtHAM | AT2G45160 | — | (Zhou et al. 2015) |
| TF 50 | WIND3 | AT1G36060 | callus | (Shin and Seo 2018) |
| TF 51 | WIND4 | AT5G65130 | callus | (Shin and Seo 2018) |
| TF 52 | BHLH148 | AT3G06590 | SAM | (Endo et al. 2016) |
| TF 53 | GL2 | AT1G79840 | ROOT HAIR | (Gaillochet and Lohmann 2015) |
| TF 54 | WOX11 | AT3G03660 | callus | (Shin and Seo 2018) |
| TF 55 | PLT3 | AT5G10510 | callus | (Shin and Seo 2018) |
| TF 56 | bZIP59 | AT2G31370 | callus | (Shin and Seo 2018) |
| TF 57 | WOX12 | AT5G17810 | callus | (Shin and Seo 2018) |
| TF 58 | PLT5 | AT5G57390 | callus | (Shin and Seo 2018) |
| TF 59 | PLT7 | AT5G65510 | callus | (Shin and Seo 2018) |
| TF 60 | ARF7 | AT5G20730 | callus | (Shin and Seo 2018) |
| TF 61 | ARF19 | AT1G19220 | callus | (Shin and Seo 2018) |
| TF 62 | ERF115 | AT5G07310 | callus | (Gaillochet and Lohmann 2015; Shin and Seo 2018) |
| TF 63 | LBD29 | AT3G58190 | callus | (Shin and Seo 2018) |
| TF 64 | PLT3 | AT5G10510 | ROOT | (Gaillochet and Lohmann 2015) |
| TF 65 | PLT2 | AT1G51190 | ROOT | (Gaillochet and Lohmann 2015) |
| TF 66 | ARR21 | AT5G07210 | callus | (Ikeuchi et al. 2013) |
| TF 67 | ESR2 | AT1G24590 | callus | (Ikeuchi et al. 2013) |
| TF 68 | RKD1 | AT1G18790 | callus | (Ikeuchi et al. 2013) |
| TF 69 | RKD2 | AT1G74480 | callus | (Ikeuchi et al. 2013) |
| TF 70 | RKD4 | AT5G53040 | callus | (Ikeuchi et al. 2013) |
| TF 71 | PLT1 | AT3G20840 | ROOT | (Gaillochet and Lohmann 2015) |
| TF 72 | BRAVO | AT5G17800 | — | (Gaillochet and Lohmann 2015) |
| TF 73 | SCR | AT3G54220 | RAM | (Gaillochet and Lohmann 2015) |
| TF 74 | BES1 | AT1G19350 | — | (Gaillochet and Lohmann 2015) |
| TF 75 | HEC1 | AT5G67060 | SHOOT | (Gaillochet and Lohmann 2015) |
| TF 76 | IPT7 | AT3G23630 | SHOOT | (Gaillochet and Lohmann 2015) |
| TF 77 | STM | AT1G62360 | SHOOT | (Gaillochet and Lohmann 2015) |
| TF 78 | SHR | AT4G37650 | RAM | (Fletcher 2020) |

1. CCGs

| **No** | **nonTF Name** | **nonTF ID** | **Organism Part** | **References** |
| --- | --- | --- | --- | --- |
| nonTF 79 | BAM7 | AT2G45880 | ROOT | (Fletcher 2020) |
| nonTF 80 | BAM8 | AT5G45300 | ROOT | (Fletcher 2020) |
| nonTF 81 | PIN1 | AT1G73590 | — | (Gaillochet and Lohmann 2015) |
| nonTF 82 | PIN3 | AT1G70940 | — | (Gaillochet and Lohmann 2015) |
| nonTF 83 | BIL1 | AT2G30980 | — | (Fletcher 2020) |
| nonTF 84 | LOG3 | AT2G37210 | SHOOT | (Gaillochet and Lohmann 2015) |
| nonTF 85 | LOG4 | AT3G53450 | SHOOT | (Gaillochet and Lohmann 2015) |
| nonTF 86 | LOG7 | AT5G06300 | SHOOT | (Gaillochet and Lohmann 2015) |
| nonTF 87 | PEPR2 | AT1G17750 | RAM | (Fletcher 2020) |
| nonTF 88 | BIN2 | AT4G18710 | — | (Fletcher 2020) |
| nonTF 89 | AHK4 | AT2G01830 | SHOOT | (Gaillochet and Lohmann 2015) |
| nonTF 90 | CKX3 | AT5G56970 | SHOOT | (Gaillochet and Lohmann 2015) |
| nonTF 91 | CKX5 | AT1G75450 | SHOOT | (Gaillochet and Lohmann 2015) |
| nonTF 92 | AHP6 | AT1G80100 | SHOOT | (Gaillochet and Lohmann 2015) |
| nonTF 93 | KRP7 | AT1G49620 | callus | (Ikeuchi et al. 2013) |
| nonTF 94 | PRC1 | AT5G64740 | callus | (Lee and Seo 2018) |
| nonTF 95 | CLE44 | AT4G13195 | — | (Song et al. 2022) |
| nonTF 96 | CLE41 | AT3G24770 | — | (Song et al. 2022) |
| nonTF 97 | ASB1 | AT1G25220 | — | (Gaillochet and Lohmann 2015) |
| nonTF 98 | CYCD3 | AT4G34160 | — | (Gaillochet and Lohmann 2015) |
| nonTF 99 | CDKB2;1 | AT1G76540 | RAM | (Gaillochet and Lohmann 2015) |
| nonTF 100 | CDKB2;2 | AT1G20930 | RAM | (Gaillochet and Lohmann 2015) |
| nonTF 101 | CDKA;1 | AT3G48750 | — | (Gaillochet and Lohmann 2015) |
| nonTF 102 | RBR | AT3G12280 | — | (Gaillochet and Lohmann 2015) |
| nonTF 103 | CLE40 | AT1G69040 | RAM | (Pallakies and Simon 2014) |
| nonTF 104 | PSK5 | AT5G65870 | ROOT | (Gaillochet and Lohmann 2015) |
| nonTF 105 | KRP3 | AT5G48820 | callus | (Ikeuchi et al. 2013) |
| nonTF 106 | CLV3 | AT2G27250 | SAM | (Hirakawa 2021) |
| nonTF 107 | KRP2 | AT3G50630 | callus | (Ikeuchi et al. 2013) |
| nonTF 108 | GCN5 | AT3G54610 | callus | (Shin and Seo 2018) |
| nonTF 109 | HAG1 | AT3G54610 | callus | (Shin and Seo 2018) |
| nonTF 110 | CAF1 | AT2G20020 | — | (Gaillochet and Lohmann 2015) |
| nonTF 111 | FAS1 | AT1G65470 | — | (Gaillochet and Lohmann 2015) |
| nonTF 112 | ATXR2 | AT3G21820 | callus | (Shin and Seo 2018) |
| nonTF 113 | GEM | AT2G22475 | — | (Gaillochet and Lohmann 2015) |
| nonTF 114 | CLV1 | AT1G75820 | SAM | (Nimchuk 2017) |
| nonTF 115 | CLV2 | AT1G75820 | SAM | (Pallakies and Simon 2014) |
| nonTF 116 | PAT1 | AT1G79090 | callus | (Shin and Seo 2018) |
| nonTF 117 | DRM1 | AT1G28330 | SAM | (Jiang et al. 2015) |
| nonTF 118 | BAM3 | AT4G17090 | LEAF | (Fletcher 2020) |
| nonTF 119 | BAM1 | AT2G40670 | LEAF | (Fletcher 2020) |
| nonTF 120 | DCL1 | AT1G01040 | — | (Garrocho-Villegas et al. 2017) |
| nonTF 121 | ARR16 | AT2G40670 | LEAF | (Fletcher 2020) |
| nonTF 122 | SERK1 | AT1G71830 | LEAF | (Fletcher 2020) |
| nonTF 123 | TSD1 | AT5G49720 | callus | (Ikeuchi et al. 2013) |
| nonTF 124 | TSD2 | AT1G78240 | callus | (Ikeuchi et al. 2013) |
| nonTF 125 | SWN | AT4G02020 | callus | (Ikeuchi et al. 2013) |
| nonTF 126 | VRN2 | AT4G16845 | callus | (Ikeuchi et al. 2013) |
| nonTF 127 | EMF2 | AT5G51230 | callus | (Ikeuchi et al. 2013) |
| nonTF 128 | FIE | AT3G20740 | callus | (Ikeuchi et al. 2013) |
| nonTF 129 | BMI1A | AT2G30580 | callus | (Ikeuchi et al. 2013) |
| nonTF 130 | BMI1B | AT1G06770 | callus | (Ikeuchi et al. 2013) |
| nonTF 131 | PKL | AT2G25170 | callus | (Ikeuchi et al. 2013) |
| nonTF 132 | OSU1, QUA2 | AT1G78240 | — | (Ikeuchi et al. 2013) |
| nonTF 133 | CLE10 | AT1G69320 | LEAF | (Fletcher 2020) |
| nonTF 134 | CIK1 | AT1G60800 | SAM | (Fletcher 2020) |
| nonTF 135 | ARR15 | AT1G74890 | SAM | (Fletcher 2020) |
| nonTF 136 | SDG2 | AT4G15180 | callus | — |
| nonTF 137 | MET1 | AT1G55480 | callus | (Shim et al. 2021) |
| nonTF 138 | CMT3 | AT1G69770 | — | (Shemer et al. 2015) |
| nonTF 139 | CLE2 | AT4G18510 | SAM | (Cheng et al. 2015) |
| nonTF 140 | GNAT1 | AT1G26220 | SAM | (Cheng et al. 2015) |
| nonTF 141 | HDA19 | AT4G38130 | Callus | (Lee and Seo 2018) |
| nonTF 142 | ARR7 | AT1G19050 | SAM | (Fletcher 2020) |
| nonTF 143 | PKR2 | AT4G31900 | Callus | (Lee and Seo 2018) |
| nonTF 144 | HDA6 | AT5G63110 | Callus | (Lee and Seo 2018) |
| nonTF 145 | ACR4 | AT1G69040 | RAM | (Motte et al. 2014) |

Aida M et al. (2020) Establishment of the embryonic shoot meristem involves activation of two classes of genes with opposing functions for meristem activities International Journal of Molecular Sciences 21:5864

Banno H, Ikeda Y, Niu Q-W, Chua N-H (2001) Overexpression of Arabidopsis ESR1 induces initiation of shoot regeneration The Plant Cell 13:2609-2618

Cheng Y et al. (2015) Down-regulation of multiple CDK inhibitor ICK/KRP genes promotes cell proliferation, callus induction and plant regeneration in Arabidopsis Frontiers in Plant Science 6:825

Du F (2020) Integrated Signals Regulate Shoot Stem Cell Homeostasis Molecular Plant 13:1535

Endo T et al. (2016) Overexpression of a citrus basic helix-loop-helix transcription factor (CubHLH1), which is homologous to Arabidopsis activation-tagged bri1 suppressor 1 interacting factor genes, modulates carotenoid metabolism in transgenic tomato Plant Science 243:35-48

Fletcher JC (2020) Recent advances in Arabidopsis CLE peptide signaling Trends in plant science 25:1005-1016

Gaillochet C, Lohmann JU (2015) The never-ending story: from pluripotency to plant developmental plasticity Development 142:2237-2249

Garrocho-Villegas V, Aguilar R, de Jiménez ES (2017) Contribution of the Zea mays insulin-like growth factor (ZmIGF) to the embryogenic competence of maize tissue cultures In Vitro Cellular & Developmental Biology-Plant 53:122-132

Hirakawa Y (2021) CLAVATA3, a plant peptide controlling stem cell fate in the meristem Peptides 142:170579

Horstman A et al. (2017) The BABY BOOM Transcription Factor Activates the LEC1-ABI3-FUS3-LEC2 Network to Induce Somatic Embryogenesis Plant Physiol 175:848-857 doi:10.1104/pp.17.00232

Ikeuchi M, Sugimoto K, Iwase A (2013) Plant callus: mechanisms of induction and repression Plant Cell 25:3159-3173 doi:10.1105/tpc.113.116053

Iwakawa H, Takahashi H, Machida Y, Machida C (2020) Roles of ASYMMETRIC LEAVES2 (AS2) and Nucleolar Proteins in the Adaxial–Abaxial Polarity Specification at the Perinucleolar Region in Arabidopsis International Journal of Molecular Sciences 21:7314

Jiang F, Xu X, Liu H, Zhu J (2015) DRM1 and DRM2 are involved in Arabidopsis callus formation Plant Cell, Tissue and Organ Culture (PCTOC) 123:221-228

Lee K, Seo PJ (2018) Dynamic epigenetic changes during plant regeneration Trends in plant science 23:235-247

Liu B et al. (2015) Histological and transcript analyses of intact somatic embryos in an elite maize (Zea mays L.) inbred line Y423 Plant Physiology and Biochemistry 92:81-91

Matsuo N, Makino M, Banno H (2011) Arabidopsis ENHANCER OF SHOOT REGENERATION (ESR) 1 and ESR2 regulate in vitro shoot regeneration and their expressions are differentially regulated Plant science 181:39-46

Motte H, Vereecke D, Geelen D, Werbrouck S (2014) The molecular path to in vitro shoot regeneration Biotechnology Advances 32:107-121

Nimchuk ZL (2017) CLAVATA1 controls distinct signaling outputs that buffer shoot stem cell proliferation through a two-step transcriptional compensation loop PLoS genetics 13:e1006681

Pallakies H, Simon R (2014) The CLE40 and CRN/CLV2 signaling pathways antagonistically control root meristem growth in Arabidopsis Molecular Plant 7:1619-1636

Sarkar AK et al. (2007) Conserved factors regulate signalling in Arabidopsis thaliana shoot and root stem cell organizers Nature 446:811-814

Shemer O, Landau U, Candela H, Zemach A, Williams LE (2015) Competency for shoot regeneration from Arabidopsis root explants is regulated by DNA methylation Plant Science 238:251-261

Shim S, Kim HK, Bae SH, Lee H, Lee HJ, Jung YJ, Seo PJ (2020) Transcriptome comparison between pluripotent and non-pluripotent calli derived from mature rice seeds Scientific reports 10:21257

Shim S, Lee HG, Seo PJ (2021) MET1-dependent DNA methylation represses light signaling and influences plant regeneration in Arabidopsis Molecules and Cells 44:746

Shimadzu S, Furuya T, Kondo Y (2023) Molecular Mechanisms Underlying the Establishment and Maintenance of Vascular Stem Cells in Arabidopsis thaliana Plant Cell Physiol 64:274-283 doi:10.1093/pcp/pcac161

Shin J, Seo PJ (2018) Varying auxin levels induce distinct pluripotent states in callus cells Frontiers in Plant Science 9:1653

Somssich M, Bleckmann A, Simon R (2016) Shared and distinct functions of the pseudokinase CORYNE (CRN) in shoot and root stem cell maintenance of Arabidopsis Journal of experimental botany 67:4901-4915

Song X-F, Hou X-L, Liu C-M (2022) CLE peptides: critical regulators for stem cell maintenance in plants Planta 255:1-17

Sugiyama M (2018) Partnership for callusing Nature Plants 4:69-70

Takahashi H et al. (2013) Meta-analyses of microarrays of Arabidopsis asymmetric leaves1 (as1), as2 and their modifying mutants reveal a critical role for the ETT pathway in stabilization of adaxial–abaxial patterning and cell division during leaf development Plant and cell physiology 54:418-431

Tao Z, Hu H, Luo X, Jia B, Du J, He Y (2019) Embryonic resetting of the parental vernalized state by two B3 domain transcription factors in Arabidopsis Nature Plants 5:424-435

Wang G, Zhang Y, Li C, Wang X, Fletcher JC (2022) Signaling peptides direct the art of rebirth Trends in Plant Science

Wang X, Niu Q-W, Teng C, Li C, Mu J, Chua N-H, Zuo J (2009) Overexpression of PGA37/MYB118 and MYB115 promotes vegetative-to-embryonic transition in Arabidopsis Cell Research 19:224-235

Xu L, Huang H (2014) Genetic and epigenetic controls of plant regeneration Current topics in developmental biology 108:1-33

Zhou Y et al. (2015) Control of plant stem cell function by conserved interacting transcriptional regulators Nature 517:377-380
